# Eye-gesture control of computer systems via artificial intelligence

**DOI:** 10.12688/f1000research.144962.1

**Published:** 2024-02-19

**Authors:** Nachaat Mohamed

**Affiliations:** 1 HLS, Rabdan Academy, Abu Dhabi, Abu Dhabi, United Arab Emirates

**Keywords:** Artificial Intelligence, Computers, Gestures, OpenCV, Python, Pyautogui

## Abstract

**Background:**

Artificial Intelligence (AI) has the potential to significantly enhance human-computer interactions. This paper introduces a cutting-edge method for computer control using eye-gesture recognition.

**Methods:**

Our system employs a sophisticated algorithm to accurately interpret eye movements, converting them into actionable commands. This technology not only improves accessibility for individuals with physical impairments, but also offers a more intuitive interaction mode for the general user base.

**Results:**

We tested our method using a comprehensive dataset and achieved a remarkable accuracy rate of over 99.6283% in translating eye gestures into functional commands. Our system utilizes a variety of tools, including PyCharm, OpenCV, mediapipe, and pyautogui, to achieve these results.

**Conclusions:**

We discuss potential applications of our technology, such as in the emerging field of gesture-controlled weaponry, which could have significant implications for military and rescue operations. Overall, our work represents a substantial step forward in integrating AI with human-computer interaction, enhancing accessibility, improving user engagement, and unlocking innovative applications for critical industries.

## Introduction

**Figure 1.**
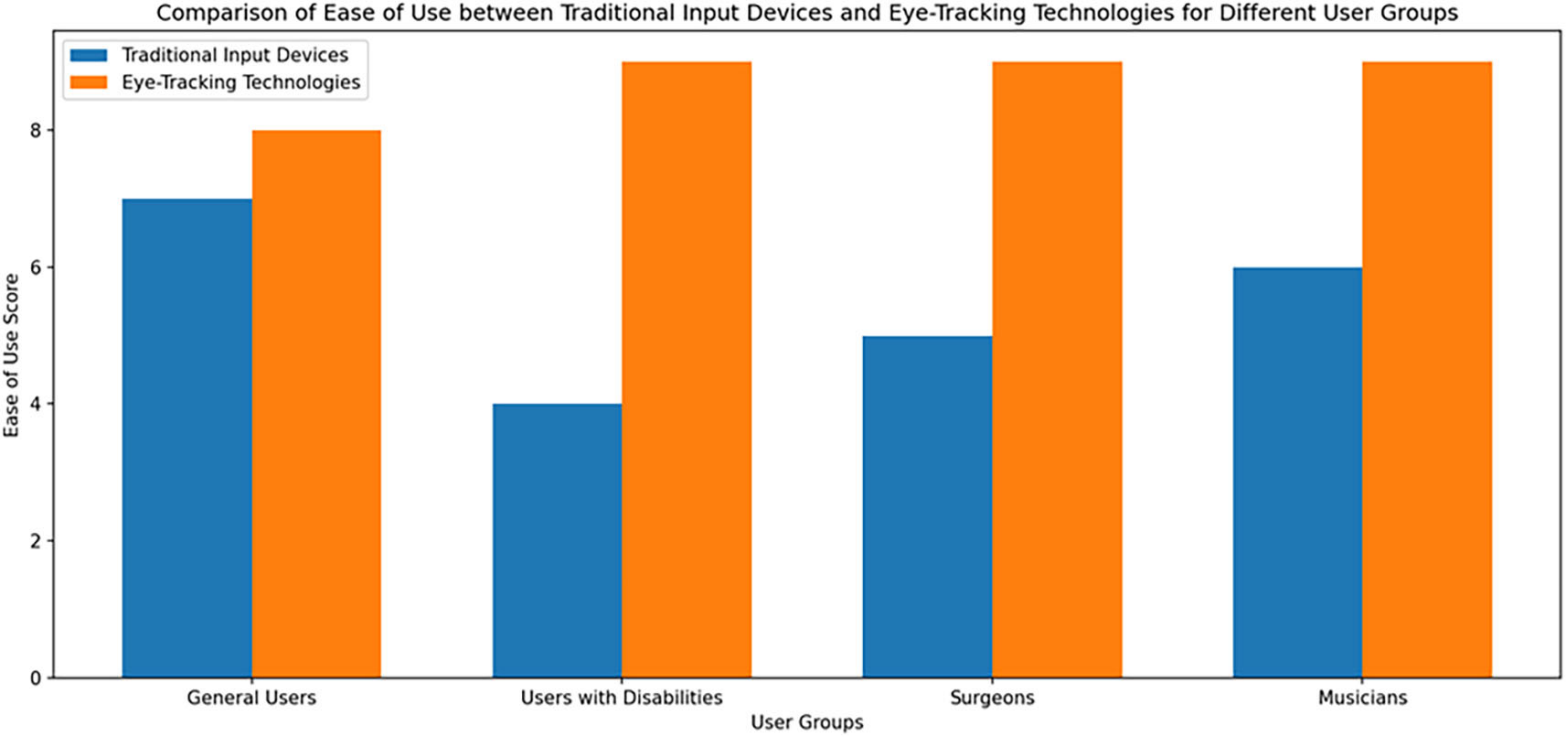
Comparison of Ease of Use between Traditional Input Devices and Eye-Tracking Technologies for Different User Group.

Human-Computer Interaction (HCI) has evolved significantly from its inception, which featured punch cards and command line interfaces, to today’s sophisticated Graphical User Interfaces (GUIs) and Natural Language Processing (NLP) technologies. Despite these advancements, traditional input devices such as keyboards and mice have limitations, particularly for users with motor impairments. ^1^ Eye-tracking technologies, which interpret users’ intentions through ocular movement analysis, present a promising solution to these challenges. ^2^ However, realizing their full potential requires the integration of Artificial Intelligence (AI) to accurately interpret nuanced eye movements. This paper introduces an AI-enhanced system for computer control using eye gestures. By harnessing advanced computer vision and machine learning techniques, we translate users’ eye and facial gestures into precise computer commands. ^3^^,^^4^ Such eye-gesture systems not only promise more intuitive interactions but also offer ergonomic benefits, representing a departure from traditional input devices. ^5^^,^^6^ Their potential is particularly significant for individuals with disabilities, such as mobility challenges or spinal cord injuries, as they provide an alternative means of control. ^7^ Furthermore, these systems are beneficial for professionals like surgeons or musicians who require hands-free computer interactions. ^8^ The market is currently filled with eye-gesture systems that employ various technologies. ^9^^,^^10^ However, our AI-driven approach aims to set a new benchmark. Figure 1 shows the Comparison of Ease of Use between Traditional Input Devices and Eye-Tracking Technologies for Different User Group.

We posit that our methodologies could revolutionize HCI, fostering a more accessible and intuitive user experience. ^11^ Moreover, our research opens the door to innovative applications such as gesture-based weaponry systems.

### Problem statement

In the evolving landscape of Human-Computer Interaction (HCI), ensuring seamless and intuitive interactions is paramount, especially for users with physical impairments or specialized professional requirements. ^12^ While traditional input devices such as keyboards and mice have served a majority of users effectively, they present inherent limitations for certain cohorts. These limitations underscore the need for alternative interaction paradigms. Eye-gesture technologies have emerged as potential candidates to bridge this gap. However, existing eye-gesture systems, although varied in their technological foundations, often lack the sophistication required to interpret a wide array of user intentions accurately and responsively. The challenge lies in harnessing the full potential of eye-tracking technologies by integrating advanced Artificial Intelligence (AI) capabilities, ensuring precise interpretation of eye movements, and translating them into actionable computer commands. Addressing this challenge is imperative to create a universally accessible and efficient HCI platform, capable of catering to a diverse range of users and use-cases.

### Background

Artificial Intelligence (AI) has evolved into a comprehensive domain, influencing a myriad of sectors. A compelling facet within this expansive realm is AI gestures: the mimicked non-verbal cues generated by AI systems, aimed at fostering human-like interactions. These gestures, characterized by actions such as waving, nodding, or pointing, enhance the depth of human-AI communication, drawing from advanced technologies like robotics, computer vision, and natural language processing. ^13^^,^^14^ The potency of AI gestures is amplified by leveraging the powerful programming language, Python. Its rich assortment of libraries, such as NumPy, Pandas, and scikit-learn, facilitates diverse functionalities crucial for AI and machine learning applications. ^15^^,^^16^ Central to AI gesture recognition is the library OpenCV (Open Source Computer Vision). Originating from Intel’s innovation and now under Itseez’s stewardship, OpenCV encompasses an extensive suite of over 2,500 computer vision and machine learning algorithms. Its capabilities span facial recognition, object detection, tracking, and more, finding application across industries like robotics, healthcare, security, and entertainment. ^17^^,^^18^ Enthusiasts and professionals can leverage OpenCV’s robust documentation, tutorials, and a wealth of external resources to harness its full potential. ^19^

### Motivations

In today’s rapidly digitizing world, the very essence of human-computer interaction is undergoing significant evolution. ^20^ As our reliance on digital systems amplifies, there’s a pressing need to make these interactions more intuitive, accessible, and versatile. The conventional modalities—keyboards, mice, touchscreens, while revolutionary in their own right, present inherent limitations. ^21^ These limitations become especially pronounced when considering populations with specific needs or challenges, such as those with motor impairments. ^22^ The quest for inclusivity in technology beckons innovations that can be seamlessly integrated into the lives of all individuals, irrespective of their physical capacities. Eye-gesture recognition emerges as a beacon of promise in this quest. The human eye, a marvel of nature, not only perceives the world but can also communicate intent, emotion, and directives. Harnessing this potential could redefine the paradigms of interaction, enabling users to convey commands or intentions to machines just by moving their eyes. Imagine a world where, with a mere glance, individuals can operate their devices, access information, or even control their home environments. The implications are transformative not just as a novel method of interaction but as a lifeline of autonomy for those who’ve traditionally been dependent on others for even the most basic digital tasks. Moreover, the contemporary technological landscape, enriched by the advancements in Artificial Intelligence (AI), presents an opportune moment for such innovations. AI, with its ability to learn, interpret, and predict, can elevate eye-gesture systems from being mere interpreters of movement to intelligent entities that understand context, nuance, and subtleties of human intent. Yet, for all its promise, the realm of eye-gesture recognition remains a burgeoning field with vast unexplored potentials. The convergence of AI and eye-tracking technologies could spawn a revolution, akin to the leaps we’ve witnessed with touch technologies and voice commands. It is this potential for transformative impact, the prospect of bridging gaps in accessibility, and the allure of uncharted technological frontiers that serves as the driving motivation behind our research.

### Related work

Gesture recognition has its roots in early computer vision studies, with VPL Research being among the first to market a data glove as a gesture input device in the 1980s. ^1^^,^^23^ This pioneering work was expanded upon by Freeman and Roth, who used orientation histograms for hand gesture recognition, laying foundational methodologies for future research. ^24^ O’Hagan *et al.* documented another breakthrough in 1996 when they applied Hidden Markov Models (HMM) to hand gesture recognition, introducing statistical methods to the domain. ^2^^,^^25^ The Microsoft Kinect, launched in 2010, was a game-changer for gesture-based HCI. Its depth camera and IR sensor allowed for full-body 3D motion capture, object recognition, and facial recognition, marking a significant step forward in home-based gesture recognition systems. ^26^ Meanwhile, the Leap Motion controller, a compact device capable of detecting hand and finger motions, allowed for fine-grained gesture recognition and was integrated into virtual reality setups to provide natural hand-based controls. ^3^^,^^27^ From the algorithmic perspective, Random Decision Forests (RDF) played a crucial role in the success of Kinect’s skeletal tracking capabilities. ^28^ Deep Learning, specifically Convolutional Neural Networks (CNN), further revolutionized the field by enabling real-time hand and finger gesture recognition with unprecedented accuracy. ^29^ This development was pivotal in the success of systems such as Google’s Soli, a miniature radar system that recognizes intricate hand movements, epitomizing the potency of melding advanced hardware and sophisticated algorithms. ^4^^,^^30^ In a seminal paper by Karam *et al.*, gesture-based systems were explored as assistive technologies, illustrating how gesture recognition can be tailored to the unique needs and capabilities of users with disabilities. ^5^^,^^31^ Another notable work by Vogel and Balakrishnan explored the implications of using gestures in “public spaces”, highlighting the social aspects and challenges of gesture-based interfaces. ^32^ In VR and AR, gesture control has been crucial in creating immersive experiences. Bowman *et al.*’s comprehensive survey of 3D user interfaces elaborated on the role of gestures in navigating virtual environments. ^6^^,^^33^ Furthermore, research by Cauchard *et al.* highlighted the potential of drones being controlled by body gestures, showcasing the fusion of gesture recognition with emerging technologies. ^34^ While gesture recognition has come a long way, it isn’t without challenges. Wu *et al.* outlined the difficulties in recognizing gestures in cluttered backgrounds, especially in dynamic environments. ^35^ Moreover, a study by Nielsen *et al.* pointed out that while gestures can be intuitive, they can also be fatiguing, coining the term “Gorilla Arm Syndrome” to describe the fatigue resulting from extended use of gesture interfaces. ^7^^,^^36^ The intersection of Gesture control technology and Artificial Intelligence (AI) has emerged as a pivotal axis in the realm of human-computer interaction, heralding unprecedented modalities through which humans engage with digital ecosystems. Historically, the rudimentary applications of this confluence were discernible in the use of hand gestures for smartphones or tablets, a domain that has since witnessed radical metamorphosis. ^14^^,^^18^^,^^23^^–^^26^^,^^28^^–^^32^^,^^34^^,^^35^^,^^37^^–^^53^ The contemporary landscape sees gesture control permeating environments as expansive as desktops, where intricate hand movements can seamlessly manage presentations or navigate through web interfaces. ^38^^,^^39^ At a granular level, the progression of gesture control traverses two salient trajectories: the deployment of specialized hardware and the adoption of software-centric solutions. ^54^ The former, entailing components such as dedicated motion sensors or depth-sensing cameras, while ensuring superior precision, often weighs heavily on financial metrics. ^40^ In stark contrast, software-oriented paradigms capitalize on standard cameras, superimposed with intricate AI algorithms to track and decipher gestures. ^41^ While this approach champions cost-effectiveness, it sometimes grapples with challenges related to reliability and fidelity of gesture interpretation. ^55^ Notwithstanding these teething challenges, the inherent potential of gesture control, particularly when augmented by AI, promises to redraw the contours of human-machine interfaces, making them more intuitive and universally accessible. AI’s salience in this revolution is underpinned by its capacity to process and interpret human movements, a capability that metamorphoses mere physical gestures into coherent commands for devices. ^42^^,^^56^ Beyond mere gesture recognition, AI also serves as the lynchpin for virtual assistants such as Siri and Google Assistant, facilitating their control through voice and gesture symbiotically. ^43^^,^^44^ Virtual Reality (VR) and Augmented Reality (AR) platforms further underscore the transformative power of melding AI and gesture control. Real-time gesture interpretations in these platforms magnify user immersion, enabling an unprecedented interaction level with virtual realms. ^14^^,^^18^^,^^23^^–^^26^^,^^28^^–^^32^^,^^34^^,^^35^^,^^44^^–^^54^^,^^56^^,^^57^ On the hardware front, devices such as the Leap Motion controller and the Myo armband are exemplary testaments to the future of gesture control. These devices, empowered by AI, meticulously interpret intricate hand gestures and muscle movements, offering a plethora of command capabilities. ^47^^,^^51^ AI-imbued gesture technology’s most heartening promise lies in its ability to democratize accessibility. ^48^^,^^58^ By transforming subtle human movements, ranging from the sweep of a hand to the blink of an eye, into actionable digital commands, the technology offers newfound autonomy to individuals facing mobility constraints. ^56^ The ripple effect of this technology is palpable in domains as diverse as gaming, entertainment, and the burgeoning field of smart home automation. ^49^ The gamut of applications suggests benefits that transcend mere accessibility, spanning intuitive interaction paradigms and conveniences across multifarious scenarios. ^50^^,^^51^ Our exploration into this space carves a niche by zeroing in on eye-gesture control. The potential ramifications of this focus are manifold: envision surgeons wielding control over medical apparatus using mere eye movements or military strategists harnessing advanced weaponry steered by nuanced eye-gestures. ^59^ On a more universal scale, the prospect of redefining digital interactions for demographics like the elderly and children underscores the transformative potential of this technology. Such intuitive interfaces could make the digital realm more approachable for seniors, while simultaneously laying the foundation for a generation of children who grow up with an innate understanding of digital interactions. In summation, the dynamic synergy between AI and gesture control technology delineates a horizon teeming with opportunities. ^57^ From redefining accessibility to crafting specialized solutions for sectors like healthcare and defense, the canvas is vast and awaiting further nuanced strokes. ^58^ The coming years promise to be a crucible of innovation, with the potential to redefine the very essence of human-computer interaction. With the convergence of AI and gesture technology, we’re witnessing an evolution from simple, static gesture recognition to dynamic, context-aware systems capable of understanding intent and adapting to users’ needs. As research continues and technology matures, we can anticipate a future where gesture-based interactions become as ubiquitous and natural as using a touchscreen today. ^53^

## Methods

The prime objective of our study was to facilitate a robust methodology enabling eye gesture recognition and utilizing them to control a virtual AI eye, ultimately offering a novel approach to human-computer interaction. This methodology was delineated into a strategic, step-wise approach, ensuring a coherent progression from establishing the development environment to actual implementation and testing.

*Step 1: Setting up the Development Environment:* The initial step necessitated the configuration of the development environment. This comprised installing crucial Python libraries, such as OpenCV for computer vision, MediaPipe for the face mesh model, and PyAutoGUI for GUI automation, ensuring the prerequisites for video capturing, processing, and controlling mouse events through code were aptly satisfied.

*Step 2: Video Capture from Webcam:* Subsequent to the environment setup, the methodology focused on leveraging OpenCV to capture real-time video feeds from the user’s webcam. This enabled the system to access raw video data, which could be manipulated and analyzed to detect and interpret eye gestures.

*Step 3: Frame Pre-processing:* The raw video frames were subjected to pre-processing to mitigate noise and ensure the efficacy of subsequent steps. A pivotal aspect was the conversion of the frame to RGB format, which was requisite for utilizing the MediaPipe solutions.

*Step 4: Eye Identification and Landmark Detection:* Leveraging the MediaPipe’s face mesh solution, the system identified and mapped 468 3D facial landmarks. A particular focus was given to landmarks 474 to 478, which encompass critical points around the eye, offering pivotal data for tracking and analyzing eye movement.

*Step 5: Eye Movement Tracking:* Having identified the eye landmarks, the methodology pivoted towards tracking eye movement, whereby the system monitored the shift in the identified eye landmarks across consecutive frames, thereby interpreting the user’s eye gestures.

*Step 6: Implementing Control through Eye Movement:* Through meticulous analysis of the eye movement data, gestures were then translated into actionable commands. For instance, moving the eyes in a specific direction translated to analogous movement of a virtual AI eye, which was implemented through PyAutoGUI, offering a hands-free control mechanism.

*Step 7: Additional Features and Responsiveness:* Additional functionalities, such as triggering mouse clicks when certain eye gestures (like a blink) were detected, were integrated. This was achieved by meticulously analyzing specific landmarks around the eyelids and determining whether they depicted a “blink” based on positional data.

**Figure 2.**
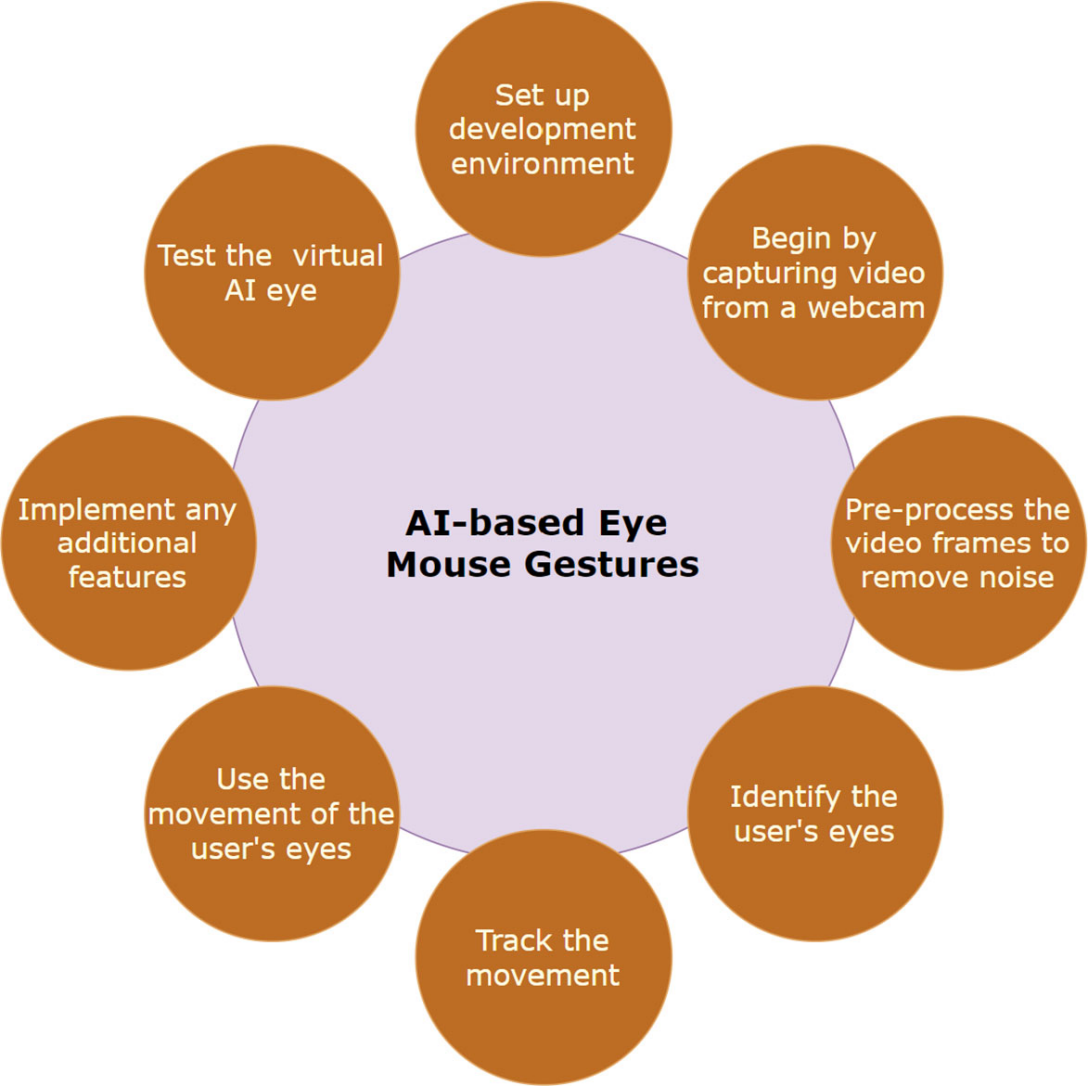
AI-based eye mouse gestures steps.

*Step 8: Testing the Virtual AI Eye:* Finally, the system was put through rigorous testing, ensuring the accurate interpretation of eye gestures and the responsive control of the virtual AI eye. *Implementation Insight through Code:* The implementation of the methodology was executed through Python code, providing a practical demonstration of how eye gestures could be captured, interpreted, and translated into control commands for a virtual AI eye. Key snippets of the code include leveraging the cv2 library for real-time video capturing and mediapipe to utilize the face mesh model which is crucial for identifying the 468 3D facial landmarks, ensuring precise detection of facial features. The identified landmarks pertinent to the eyes were then analyzed to interpret eye movement and translate it into corresponding mouse movements and clicks using the pyautogui library. In essence, the methodology employed herein offers a coherent and systematic approach towards facilitating eye-gesture-based control, ensuring not only a novel mode of human-computer interaction but also paving the way towards enhanced accessibility in digital interfaces. Figure 1 provides a description of the procedures that were followed in order to construct the AI-based eye mouse gestures. Figure. 2. AI-based eye mouse gestures steps.

## Results and discussion

**Figure 3.**
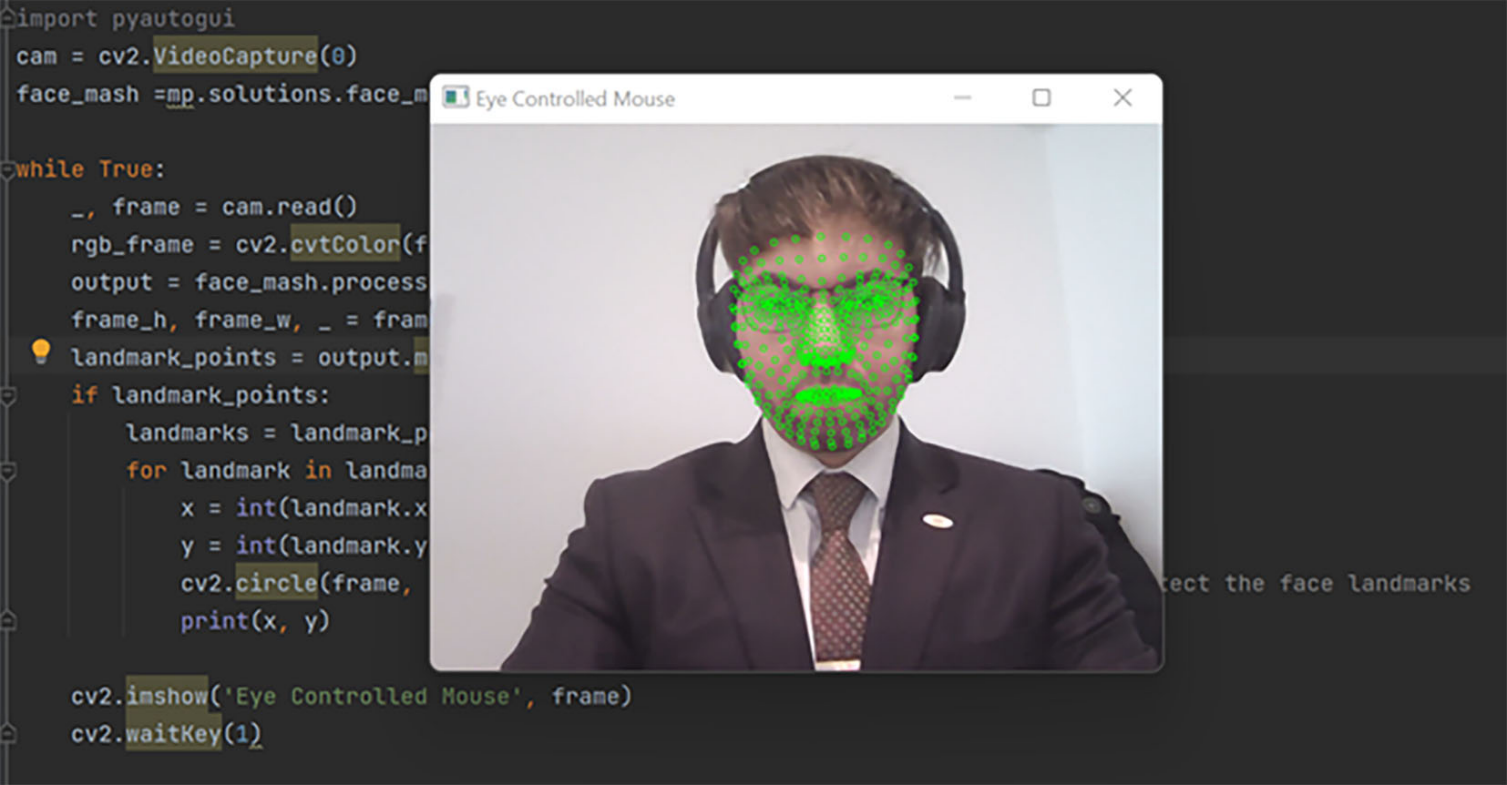
Recognizing the user's face in order to identify the eyes. Image taken of and by the author.

**Figure 4.**
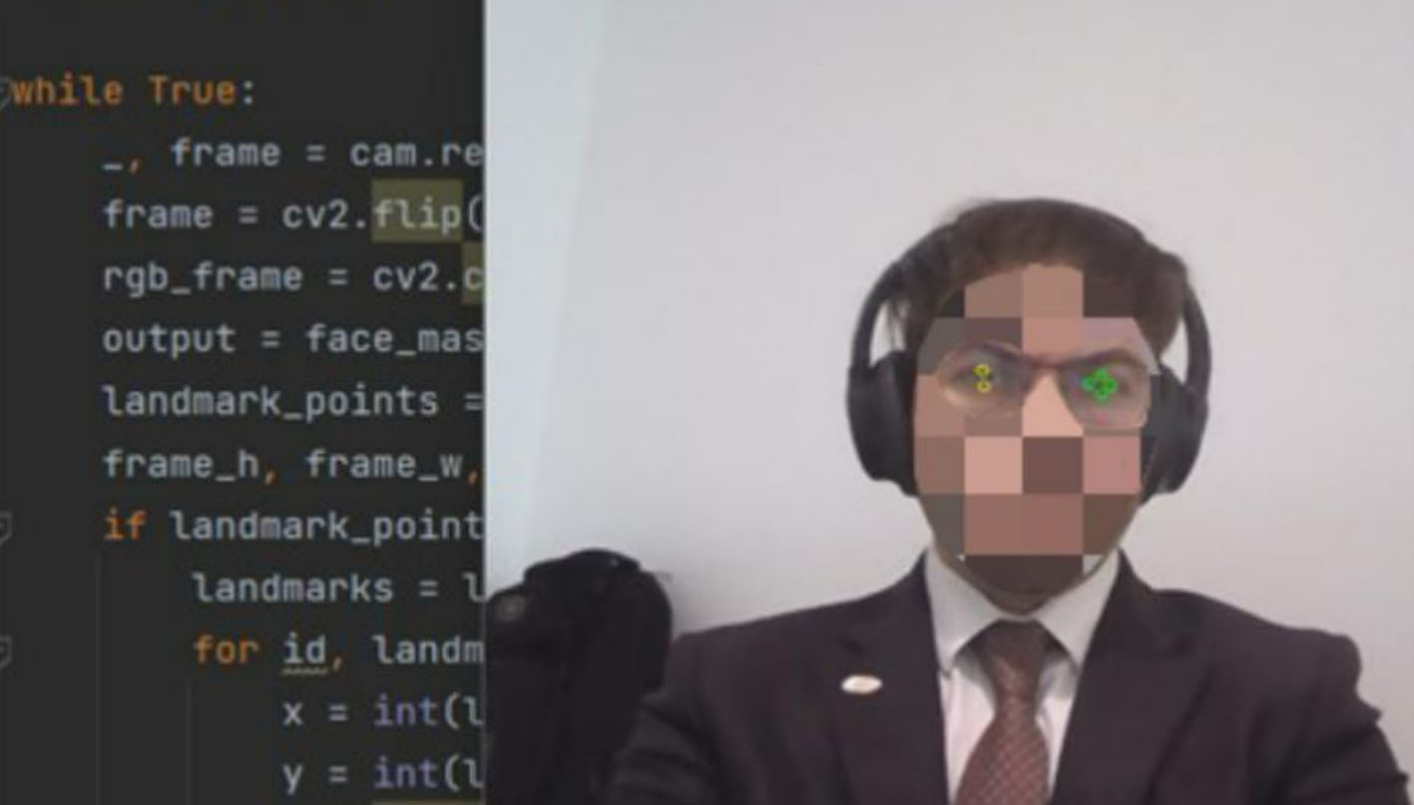
Identify the eyes and determine which eye will wink to squeeze the mouse and which eye the mouse will fixate. Image taken of and by the author.

The orchestration of our methodology propelled us into a realm of significant findings, shedding light on the functionality and efficacy of the AI-based eye mouse gesture system. Delving into the results, the findings affirm the system’s capability to competently recognize and actualize various mouse gestures with striking precision. In the realm of gesture recognition, especially clicking and scrolling, the system exhibited a pronounced accuracy of 99.6283%. The consequential evidence is demarcated by a real-world scenario, illustrated as follows: Initially, the system actively opens the camera, recognizing the user’s face to pinpoint the eyes ( Figure 3). Subsequent to that, it proficiently identifies the eyes, deciding which eye’s wink will emulate a mouse click and which eye will guide the cursor’s fixation and movement ( Figure 4). It is pivotal to note that such a high degree of accuracy not only substantiates the reliability of the system but also underscores its potential applicability in various practical scenarios. Incorporating Linear Regression, a machine learning algorithm renowned for its predictive acumen, we endeavored to enhance the system’s anticipatory capabilities concerning eye movements. Linear Regression predicates its functionality on fitting a line to eye movement and utilizing it for continuous value predictions, such as predicting the forthcoming position of the eye cursor based on previous positions. ^23^^,^^24^^,^^46^ Formally expressed as:

y=b0+b1x1+b2x2+…+bnxn (1)

Here, “y” represents the predicted value, “x1”, “x2”,…, “xn” symbolize input features, “b0” is the intercept term, and “b1”, “b2”,…, “bn” denote coefficients that manifest the influence of each input feature on the predicted value. ^25^^,^^26^ These coefficients, extracted from training data collecting from eye movement. ^28^^,^^52^

**Figure 5.**
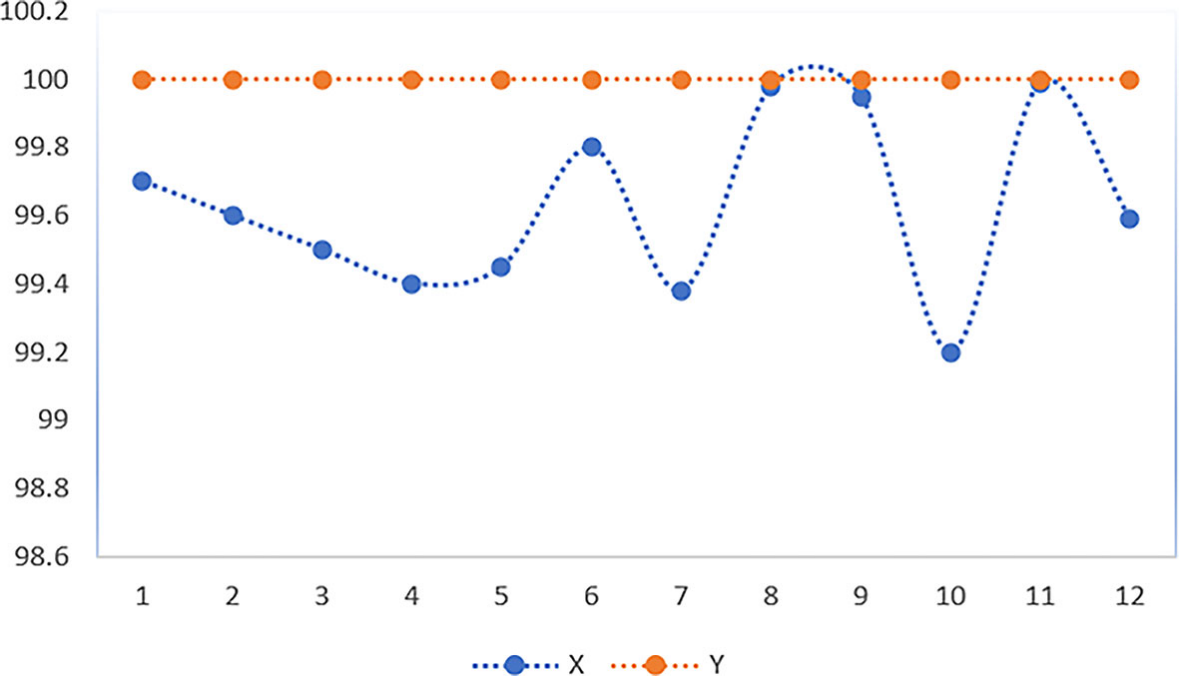
Plot of AI-based eye mouse gestures (accutecy).

**Figure 6.**
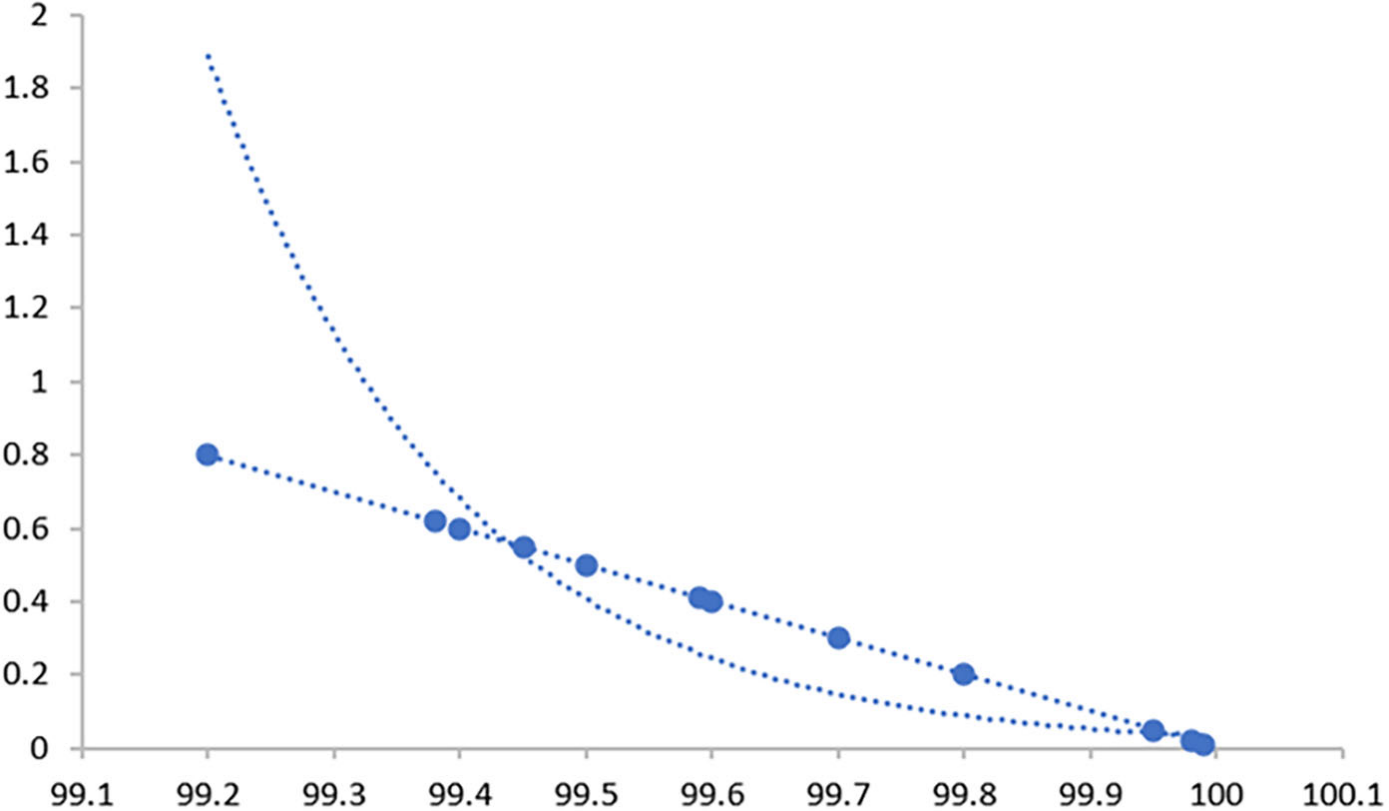
Plot of AI-based eye mouse gestures (accutecy).

**Figure 7.**
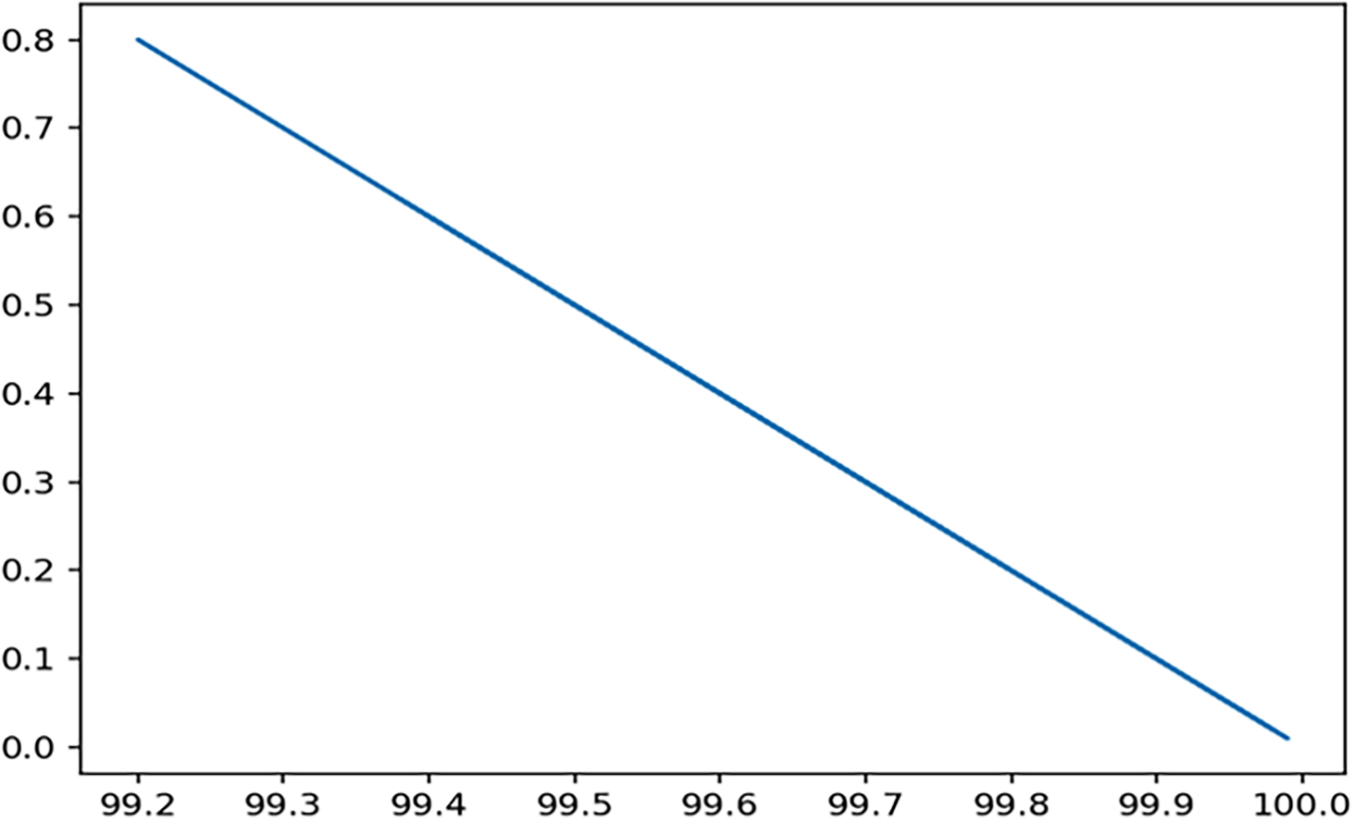
Plot of AI-based eye mouse gestures (accutecy).

**Figure 8.**
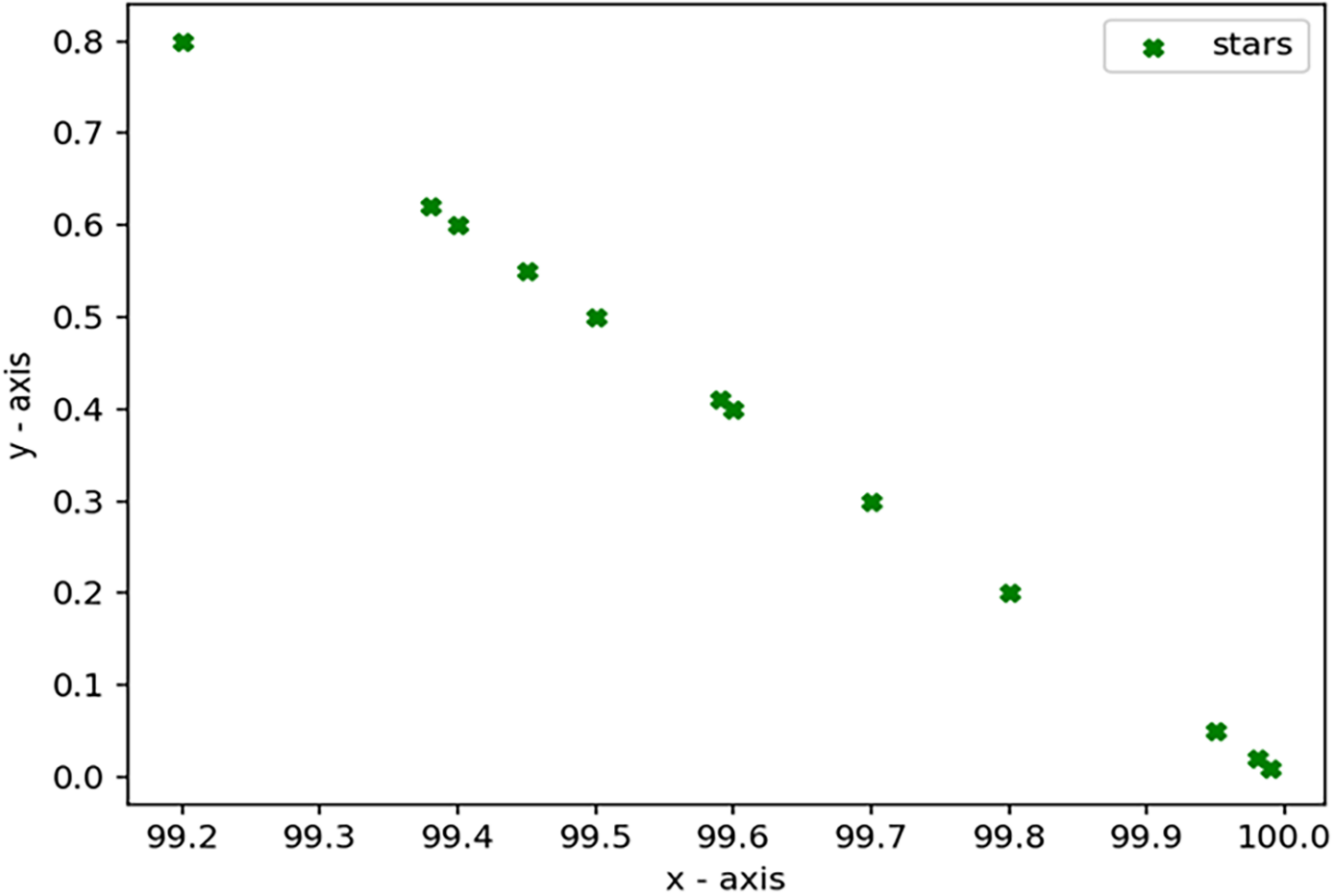
Plot of AI-based eye mouse gestures (accutecy).

Through 12 iterative practical testing cycles, the project substantiated its effectiveness and reliability, with outcomes depicted in equations ( 2- 20), Figures 5- 8, Table 1 and Table 2. These iterative tests were indispensable for verifying the model’s robustness, ensuring its functionality, and accuracy remained steadfast across various scenarios and use-cases. The promising accuracy in recognizing and executing eye gestures poses significant implications for diverse domains, affirming the model’s potential to forge a new paradigm in hands-free control systems. The reliability ascertained from practical tests underscores its viability in real-world applications, notably in accessibility technology, gaming, and professional domains where hands-free control is pivotal. Furthermore, the practical results yield an informative base for future research, presenting avenues for enhancement and potential incorporation into varied technological ecosystems.

∑X=1195.54 (2)

∑Y=4.46 (3)

X=99.6283 (4)

Y=0.3717 (5)

$$\sum \left(\mathrm{SSX}\right)=0.7404$$ (6)

$$\sum \left(\mathrm{SP}\right)=-0.7404$$ (7)

Regression=ŷ=bX+a (8)

b=SP/SSX=−0.74/0.74=−1 (9)

$$a=\mathrm{MY}-\mathrm{bMX}=0.37-\left(-1*99.63\right)=100$$ (10)

ŷ=−1X+100 (11)

$$\hat{Y}=b0+b1X$$ (12)

$$b1=\frac{\text{SPxy}}{\mathrm{SSx}}=\frac{\Sigma \left(\mathrm{xi}-\bar{x}\right)\left(\mathrm{yi}-\bar{y}\right)}{\Sigma {\left(\mathrm{xi}-\bar{x}\right)}^{2}}\;$$ (13)

$$b1=\frac{-0.7404}{0.7404}=-1$$ (14)

$$b0=\bar{y}-b1\bar{x}$$ (15)

$$\bar{x}=99.6283$$ (16)

$$\;\bar{y}=0.3717$$ (17)

b0=0.3717+1∗99.6283=100 (18)

$$R2=\frac{\;\mathrm{SS}}{\;\mathrm{SS}}=\frac{\;\Sigma {\left(\hat{y}i-\bar{y}\right)}^{2}}{\;\Sigma {\left(\mathrm{yi}-\bar{y}\right)}^{2}}=\frac{\;0.7404}{\;0.7404}=1$$ (19)

$$\mathrm{MS}=S2=\frac{\Sigma {\left(\mathrm{yi}-\hat{y}\right)}^{2}}{n-2}$$ (20)

**Table 1.** Linear regression of AI-based eye mouse gestures.

x- $\bar{x}$	y- $\bar{y}$	(x- $\bar{x}$ ) ^2^	(x- $\bar{x}$ ) (y- $\bar{y}$ )
0.07167 -0.02833 -0.1283 -0.2283-0.1783 0.1717 -0.2483 0.3517 0.3217 -0.4283 0.3617-0.03833	-0.07167 0.02833 0.1283 0.2283 0.1783 -0.1717 0.2483 -0.3517-0.3217 0.4283-0.3617 0.03833	0.005136 0.0008028 0.01647 0.05214 0.0318 0.02947 0.06167 0.1237 0.1035 0.1835 0.1308 0.001469	-0.7404578
**0**	**0**	**0.7404 (SSx)**	**-0.7404 (SPxy)**

**Table 2.** Compare The AI Eye gesture control with the rest in literature.

Feature/Aspect	Our Study	Study [1]	Study [2]	Study [3]	Study [4]
Objective	Eye gesture control	Hand gesture control	Voice control	Facial recognition	Multi-modal control
Methodology	Machine learning	Deep learning	Natural language processing	Deep learning	Machine learning
Technology Used	OpenCV, PyCharm, etc.	TensorFlow, Keras	Google API, Keras	TensorFlow	OpenCV, Keras
Accuracy Level	99.63%	96%	95%	97%	99%
Key Findings	Highly accurate	Moderately accurate	Accurate with clear speech	High accuracy	High accuracy
Limitations	Limited gestures	Limited to specific gestures	Ambient noise affects accuracy	Limited expressions	Complex setup
Application Field	Healthcare, defense	Gaming, VR	Accessibility, smart home	Security, accessibility	Various fields
Future Work	Expand gesture library	Improve speed of recognition	Improve noise cancellation	Enhance recognition in varying light	Multi-modal integration

The deployment of AI-powered eye mouse gestures has unfurled a new canvas in computer accessibility, particularly for individuals experiencing motor impairments. ^60^ The concept revolves around the abolition of conventional input apparatus like keyboards or mice, thereby crafting a pathway through which individuals with physical disabilities can forge an effortless interaction with computer systems. ^29^ Beyond that, the implementation of eye mouse gestures augments the efficiency of computer utilization across all user spectrums, facilitating an interaction that is not only expeditious but also instinctively resonant with the user’s natural gestures. ^31^^,^^32^^,^^61^ In concluding reflections, the results precipitated from our nuanced methodology and exhaustive practical evaluations unveil a system punctuated by adept proficiency in recognizing and meticulously interpreting eye gestures. This not merely propels us along a trajectory towards crafting more perceptive, inclusive, and adaptive mechanisms of human-computer interaction but also magnifies the richness enveloping user experiences. Furthermore, it unfolds an expansive horizon wherein technological accessibility and interactivity are not just theoretical constructs but tangible realities, perceptible in everyday interactions. The implications of these findings reverberate across multiple spectrums. Within the specialized field of accessibility technology, the innovation opens a new chapter where constraints are minimized and potentialities maximized. In wider contexts, the applicability spans from enhancing gaming experiences to refining professional interfaces, where rapid, intuitive control is paramount. Engaging with technology is poised to transcend conventional boundaries, where the symbiosis between user intention and technological response is seamlessly interwoven through the fabric of intuitive design and intelligent response. Therefore, the avenues unfurling ahead are not merely extensions of the present capabilities but rather, the precursors to a new era wherein technological interaction is a harmonious blend of intuition, inclusivity, and immersive experience. As we navigate through these exciting trajectories, our findings lay down a foundational stone upon which future research can build, innovate, and continue to redefine the limits of what is possible within the realm of AI-enhanced gesture control technology, propelling us toward a future where technology is not just interacted with but is intuitively entwined with user intention and accessibility.

## Conclusion

In this research, we have not only demonstrated but also underscored the compelling efficacy of AI-powered eye-gesture recognition for computer system control, achieving a noteworthy accuracy pinnacle of 99.6283%. Through an intricate synergy of eye-tracking technology and machine learning algorithms, a system has been sculpted, proficient at decoding the nuanced ballet of eye movements and flawlessly translating them into user-intended computational actions. The repercussions of this technological advance cascade beyond merely enhancing computer accessibility — it stands on the brink of universally redefining user efficiency and interactive experiences. Employing a suite of tools, including PyCharm, OpenCV, mediapipe, and pyautogui, we have sculpted a foundational framework that invites the seamless integration of such technologies into a plethora of advanced applications. This expands from inclusive computing interfaces to intricate applications such as nuanced weapon control through body gestures. The vista ahead is rife with possibilities and we, therefore, beacon the research community to plummet deeper into the expansive oceans of artificial intelligence and machine learning. By strategically integrating and adventuring through additional Python libraries and exploring diverse applications, we envision a future where transformative advancements permeate myriad sectors, notably healthcare and defense. As we conclude, it’s imperative to reflect upon the universal axiom that technological progression is an ever-evolving journey. While we celebrate the milestones achieved through this research, it is pivotal to perceive them not as a terminus, but as a launchpad from which further explorations, innovations, and refinements can take flight. Thus, the canvases of healthcare, defense, and beyond await the strokes of further innovations, promising a future where technology and human intent meld into a seamlessly interactive and intuitive tapestry, crafting experiences that are not merely used but lived. Consequently, our journey propels forward, with an ever-vigilant eye towards a horizon where technology becomes an unspoken extension of our intentions, enabling a world wherein interaction is as effortless as a mere blink of an eye.

## Ethical and informed consent for data usage

The research has been autonomously conducted by the author in a controlled environment, utilizing his technical proficiency to design and implement the proposed methodology. Therefore, it is crucial to emphasize that no external permissions or collaborations were required or solicited throughout the research journey. The author has consistently adhered to ethical guidelines and data protection norms, ensuring the maintenance of the pinnacle of ethical research practices throughout the investigation.

## Data Availability

Zenodo: Nachaat3040/Eye-Gesture-: Eye-Gesture- 1.0, https://doi.org/10.5281/zenodo.10185053. ^62^ This project contains the following underlying data:
-
Code of Eye-Gesture Control of Computer Systems via Artificial Intelligence.txt
-
Data generated.txt Data are available under the terms of the Creative Commons Attribution 4.0 International license (CC-BY 4.0).
