# Eye-gesture control of computer systems via artificial intelligence

**DOI:** 10.12688/f1000research.144962.2

**Published:** 2024-12-18

**Authors:** Nachaat Mohamed

**Affiliations:** 1 HLS, Rabdan Academy, Abu Dhabi, Abu Dhabi, United Arab Emirates

**Keywords:** Artificial Intelligence, Computers, Gestures, OpenCV, Python, Pyautogui

## Abstract

**Background:**

Artificial Intelligence (AI) offers transformative potential for human-computer interaction, particularly through eye-gesture recognition, enabling intuitive control for users and accessibility for individuals with physical impairments.

**Methods:**

We developed an AI-driven eye-gesture recognition system using tools like OpenCV, MediaPipe, and PyAutoGUI to translate eye movements into commands. The system was trained on a dataset of 20,000 gestures from 100 diverse volunteers, representing various demographics, and tested under different conditions, including varying lighting and eyewear.

**Results:**

The system achieved 99.63% accuracy in recognizing gestures, with slight reductions to 98.9% under reflective glasses. These results demonstrate its robustness and adaptability across scenarios, confirming its generalizability.

**Conclusions:**

This system advances AI-driven interaction by enhancing accessibility and unlocking applications in critical fields like military and rescue operations. Future work will validate the system using publicly available datasets to further strengthen its impact and usability.

## Introduction

**Figure 1.**
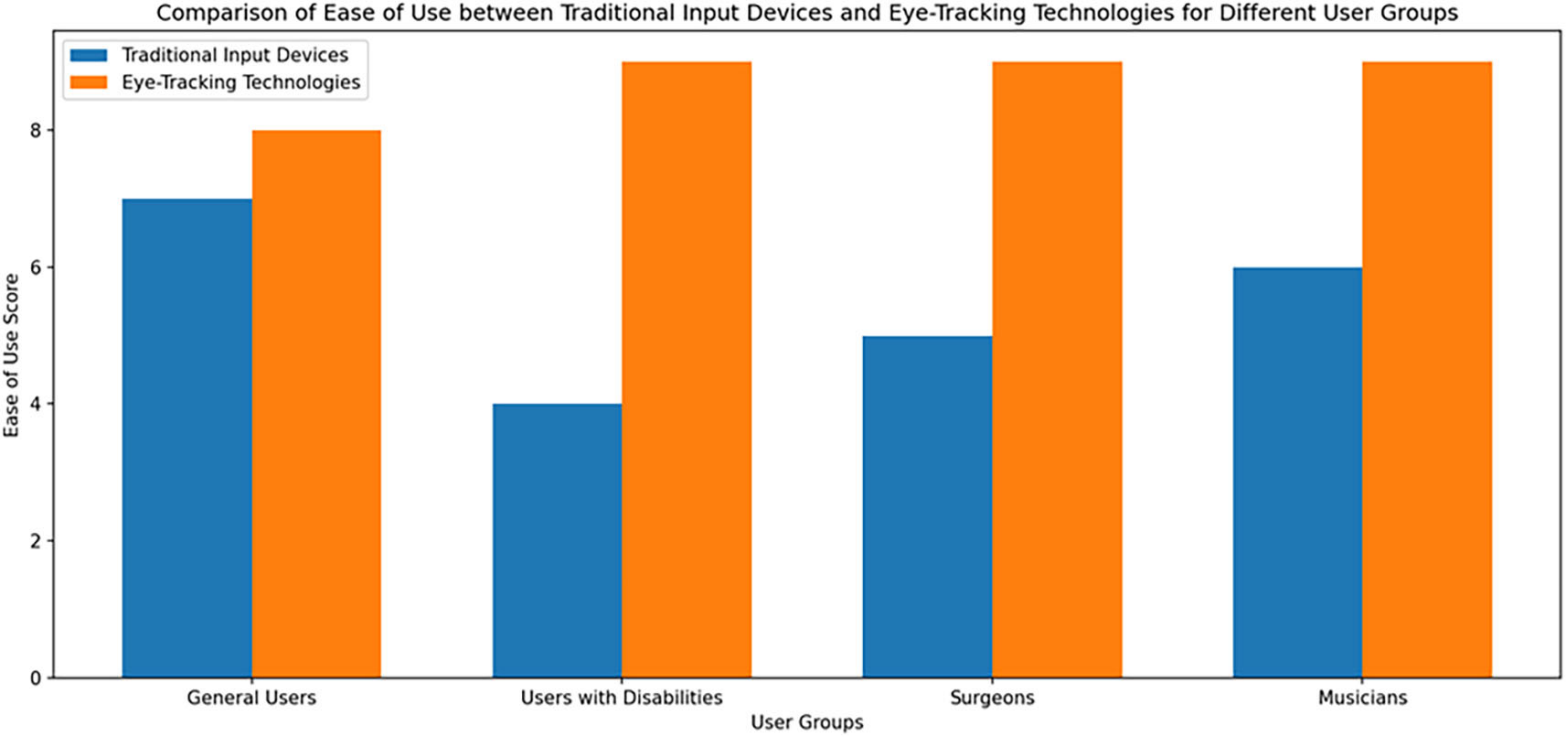
Comparison of Ease of Use between Traditional Input Devices and Eye-Tracking Technologies for Different User Group.

Human-Computer Interaction (HCI) has evolved significantly from its inception, which featured punch cards and command line interfaces, to today’s sophisticated Graphical User Interfaces (GUIs) and Natural Language Processing (NLP) technologies. Despite these advancements, traditional input devices such as keyboards and mice have limitations, particularly for users with motor impairments. ^1^ Eye-tracking technologies, which interpret users’ intentions through ocular movement analysis, present a promising solution to these challenges. ^2^ However, realizing their full potential requires the integration of Artificial Intelligence (AI) to accurately interpret nuanced eye movements. This paper introduces an AI-enhanced system for computer control using eye gestures. By harnessing advanced computer vision and machine learning techniques, we translate users’ eye and facial gestures into precise computer commands. ^3^^,^^4^ Such eye-gesture systems not only promise more intuitive interactions but also offer ergonomic benefits, representing a departure from traditional input devices. ^5^^,^^6^ Their potential is particularly significant for individuals with disabilities, such as mobility challenges or spinal cord injuries, as they provide an alternative means of control. ^7^ Furthermore, these systems are beneficial for professionals like surgeons or musicians who require hands-free computer interactions. ^8^ The market is currently filled with eye-gesture systems that employ various technologies. ^9^^,^^10^ However, our AI-driven approach aims to set a new benchmark. Figure 1 shows the Comparison of Ease of Use between Traditional Input Devices and Eye-Tracking Technologies for Different User Group.

We posit that our methodologies could revolutionize HCI, fostering a more accessible and intuitive user experience. ^11^ Moreover, our research opens the door to innovative applications such as gesture-based weaponry systems.

### Problem statement

In the evolving landscape of Human-Computer Interaction (HCI), ensuring seamless and intuitive interactions is paramount, especially for users with physical impairments or specialized professional requirements. ^12^ While traditional input devices such as keyboards and mice have served a majority of users effectively, they present inherent limitations for certain cohorts. These limitations underscore the need for alternative interaction paradigms. Eye-gesture technologies have emerged as potential candidates to bridge this gap. However, existing eye-gesture systems, although varied in their technological foundations, often lack the sophistication required to interpret a wide array of user intentions accurately and responsively. The challenge lies in harnessing the full potential of eye-tracking technologies by integrating advanced Artificial Intelligence (AI) capabilities, ensuring precise interpretation of eye movements, and translating them into actionable computer commands. Addressing this challenge is imperative to create a universally accessible and efficient HCI platform, capable of catering to a diverse range of users and use-cases.

### Background

Artificial Intelligence (AI) has evolved into a comprehensive domain, influencing a myriad of sectors. A compelling facet within this expansive realm is AI gestures: the mimicked non-verbal cues generated by AI systems, aimed at fostering human-like interactions. These gestures, characterized by actions such as waving, nodding, or pointing, enhance the depth of human-AI communication, drawing from advanced technologies like robotics, computer vision, and natural language processing. ^13^^,^^14^ The potency of AI gestures is amplified by leveraging the powerful programming language, Python. Its rich assortment of libraries, such as NumPy, Pandas, and scikit-learn, facilitates diverse functionalities crucial for AI and machine learning applications. ^15^^,^^16^ Central to AI gesture recognition is the library OpenCV (Open Source Computer Vision). Originating from Intel’s innovation and now under Itseez’s stewardship, OpenCV encompasses an extensive suite of over 2,500 computer vision and machine learning algorithms. Its capabilities span facial recognition, object detection, tracking, and more, finding application across industries like robotics, healthcare, security, and entertainment. ^17^^,^^18^ Enthusiasts and professionals can leverage OpenCV’s robust documentation, tutorials, and a wealth of external resources to harness its full potential. ^19^

### Motivations

In today’s rapidly digitizing world, the very essence of human-computer interaction is undergoing significant evolution. ^20^ As our reliance on digital systems amplifies, there’s a pressing need to make these interactions more intuitive, accessible, and versatile. The conventional modalities—keyboards, mice, touchscreens, while revolutionary in their own right, present inherent limitations. ^21^ These limitations become especially pronounced when considering populations with specific needs or challenges, such as those with motor impairments. ^22^ The quest for inclusivity in technology beckons innovations that can be seamlessly integrated into the lives of all individuals, irrespective of their physical capacities. Eye-gesture recognition emerges as a beacon of promise in this quest. The human eye, a marvel of nature, not only perceives the world but can also communicate intent, emotion, and directives. Harnessing this potential could redefine the paradigms of interaction, enabling users to convey commands or intentions to machines just by moving their eyes. Imagine a world where, with a mere glance, individuals can operate their devices, access information, or even control their home environments. The implications are transformative not just as a novel method of interaction but as a lifeline of autonomy for those who’ve traditionally been dependent on others for even the most basic digital tasks. Moreover, the contemporary technological landscape, enriched by the advancements in Artificial Intelligence (AI), presents an opportune moment for such innovations. AI, with its ability to learn, interpret, and predict, can elevate eye-gesture systems from being mere interpreters of movement to intelligent entities that understand context, nuance, and subtleties of human intent. Yet, for all its promise, the realm of eye-gesture recognition remains a burgeoning field with vast unexplored potentials. The convergence of AI and eye-tracking technologies could spawn a revolution, akin to the leaps we’ve witnessed with touch technologies and voice commands. It is this potential for transformative impact, the prospect of bridging gaps in accessibility, and the allure of uncharted technological frontiers that serves as the driving motivation behind our research.

### Related work

Gesture recognition has its roots in early computer vision studies, with VPL Research being among the first to market a data glove as a gesture input device in the 1980s. ^1^^,^^23^ This pioneering work was expanded upon by Freeman and Roth, who used orientation histograms for hand gesture recognition, laying foundational methodologies for future research. ^24^ O’Hagan *et al.* documented another breakthrough in 1996 when they applied Hidden Markov Models (HMM) to hand gesture recognition, introducing statistical methods to the domain. ^2^^,^^25^ The Microsoft Kinect, launched in 2010, was a game-changer for gesture-based HCI. Its depth camera and IR sensor allowed for full-body 3D motion capture, object recognition, and facial recognition, marking a significant step forward in home-based gesture recognition systems. ^26^ Meanwhile, the Leap Motion controller, a compact device capable of detecting hand and finger motions, allowed for fine-grained gesture recognition and was integrated into virtual reality setups to provide natural hand-based controls. ^3^^,^^27^ From the algorithmic perspective, Random Decision Forests (RDF) played a crucial role in the success of Kinect’s skeletal tracking capabilities. ^28^ Deep Learning, specifically Convolutional Neural Networks (CNN), further revolutionized the field by enabling real-time hand and finger gesture recognition with unprecedented accuracy. ^29^ This development was pivotal in the success of systems such as Google’s Soli, a miniature radar system that recognizes intricate hand movements, epitomizing the potency of melding advanced hardware and sophisticated algorithms. ^4^^,^^30^ In a seminal paper by Karam *et al.*, gesture-based systems were explored as assistive technologies, illustrating how gesture recognition can be tailored to the unique needs and capabilities of users with disabilities. ^5^^,^^31^ Another notable work by Vogel and Balakrishnan explored the implications of using gestures in “public spaces”, highlighting the social aspects and challenges of gesture-based interfaces. ^32^ In VR and AR, gesture control has been crucial in creating immersive experiences. Bowman *et al.*’s comprehensive survey of 3D user interfaces elaborated on the role of gestures in navigating virtual environments. ^6^^,^^33^ Furthermore, research by Cauchard *et al.* highlighted the potential of drones being controlled by body gestures, showcasing the fusion of gesture recognition with emerging technologies. ^34^ While gesture recognition has come a long way, it isn’t without challenges. Wu *et al.* outlined the difficulties in recognizing gestures in cluttered backgrounds, especially in dynamic environments. ^35^ Moreover, a study by Nielsen *et al.* pointed out that while gestures can be intuitive, they can also be fatiguing, coining the term “Gorilla Arm Syndrome” to describe the fatigue resulting from extended use of gesture interfaces. ^7^^,^^36^ The intersection of Gesture control technology and Artificial Intelligence (AI) has emerged as a pivotal axis in the realm of human-computer interaction, heralding unprecedented modalities through which humans engage with digital ecosystems. Historically, the rudimentary applications of this confluence were discernible in the use of hand gestures for smartphones or tablets, a domain that has since witnessed radical metamorphosis. ^14^^,^^18^^,^^23^^–^^26^^,^^28^^–^^32^^,^^34^^,^^35^^,^^37^^–^^53^ The contemporary landscape sees gesture control permeating environments as expansive as desktops, where intricate hand movements can seamlessly manage presentations or navigate through web interfaces. ^38^^,^^39^ At a granular level, the progression of gesture control traverses two salient trajectories: the deployment of specialized hardware and the adoption of software-centric solutions. ^54^ The former, entailing components such as dedicated motion sensors or depth-sensing cameras, while ensuring superior precision, often weighs heavily on financial metrics. ^40^ In stark contrast, software-oriented paradigms capitalize on standard cameras, superimposed with intricate AI algorithms to track and decipher gestures. ^41^ While this approach champions cost-effectiveness, it sometimes grapples with challenges related to reliability and fidelity of gesture interpretation. ^55^ Notwithstanding these teething challenges, the inherent potential of gesture control, particularly when augmented by AI, promises to redraw the contours of human-machine interfaces, making them more intuitive and universally accessible. AI’s salience in this revolution is underpinned by its capacity to process and interpret human movements, a capability that metamorphoses mere physical gestures into coherent commands for devices. ^42^^,^^56^ Beyond mere gesture recognition, AI also serves as the lynchpin for virtual assistants such as Siri and Google Assistant, facilitating their control through voice and gesture symbiotically. ^43^^,^^44^ Virtual Reality (VR) and Augmented Reality (AR) platforms further underscore the transformative power of melding AI and gesture control. Real-time gesture interpretations in these platforms magnify user immersion, enabling an unprecedented interaction level with virtual realms. ^14^^,^^18^^,^^23^^–^^26^^,^^28^^–^^32^^,^^34^^,^^35^^,^^44^^–^^54^^,^^56^^,^^57^ On the hardware front, devices such as the Leap Motion controller and the Myo armband are exemplary testaments to the future of gesture control. These devices, empowered by AI, meticulously interpret intricate hand gestures and muscle movements, offering a plethora of command capabilities. ^47^^,^^51^ AI-imbued gesture technology’s most heartening promise lies in its ability to democratize accessibility. ^48^^,^^58^ By transforming subtle human movements, ranging from the sweep of a hand to the blink of an eye, into actionable digital commands, the technology offers newfound autonomy to individuals facing mobility constraints. ^56^ The ripple effect of this technology is palpable in domains as diverse as gaming, entertainment, and the burgeoning field of smart home automation. ^49^ The gamut of applications suggests benefits that transcend mere accessibility, spanning intuitive interaction paradigms and conveniences across multifarious scenarios. ^50^^,^^51^ Our exploration into this space carves a niche by zeroing in on eye-gesture control. The potential ramifications of this focus are manifold: envision surgeons wielding control over medical apparatus using mere eye movements or military strategists harnessing advanced weaponry steered by nuanced eye-gestures. ^59^ On a more universal scale, the prospect of redefining digital interactions for demographics like the elderly and children underscores the transformative potential of this technology. Such intuitive interfaces could make the digital realm more approachable for seniors, while simultaneously laying the foundation for a generation of children who grow up with an innate understanding of digital interactions. In summation, the dynamic synergy between AI and gesture control technology delineates a horizon teeming with opportunities. ^57^ From redefining accessibility to crafting specialized solutions for sectors like healthcare and defense, the canvas is vast and awaiting further nuanced strokes. ^58^ The coming years promise to be a crucible of innovation, with the potential to redefine the very essence of human-computer interaction. With the convergence of AI and gesture technology, we’re witnessing an evolution from simple, static gesture recognition to dynamic, context-aware systems capable of understanding intent and adapting to users’ needs. As research continues and technology matures, we can anticipate a future where gesture-based interactions become as ubiquitous and natural as using a touchscreen today. ^53^

In comparison to existing eye-gesture control technologies, our system achieves a significantly higher accuracy of 99.63%. Prior systems, as reported in the literature, typically demonstrate accuracies ranging from 95% to 99%. However, many of these systems require specialized hardware or rely on algorithms that struggle to maintain robustness under real-world conditions, such as varying lighting or user-specific differences. Our system stands out by utilizing readily available and widely recognized tools, such as OpenCV and PyAutoGUI, which enable precise eye-movement detection and seamless command execution. This approach eliminates the need for specialized hardware, making the system more accessible and cost-effective. Furthermore, the integration of advanced machine learning models enhances its adaptability to diverse demographics and scenarios, ensuring consistent performance even in challenging conditions. By addressing limitations commonly faced by existing technologies, such as slow response times and reduced accuracy in dynamic environments, our system offers a scalable, practical, and highly accurate solution for real-time eye-gesture control. This combination of simplicity, cost-effectiveness, and high performance represents a significant advancement in the field.

## Methods

The prime objective of our study was to facilitate a robust methodology enabling eye gesture recognition and utilizing them to control a virtual AI eye, ultimately offering a novel approach to human-computer interaction. This methodology was delineated into a strategic, step-wise approach, ensuring a coherent progression from establishing the development environment to actual implementation and testing.

*Step 1: Setting up the Development Environment:* The initial step necessitated the configuration of the development environment. This comprised installing crucial Python libraries, such as OpenCV for computer vision, MediaPipe for the face mesh model, and PyAutoGUI for GUI automation, ensuring the prerequisites for video capturing, processing, and controlling mouse events through code were aptly satisfied.

*Step 2: Video Capture from Webcam:* Subsequent to the environment setup, the methodology focused on leveraging OpenCV to capture real-time video feeds from the user’s webcam. This enabled the system to access raw video data, which could be manipulated and analyzed to detect and interpret eye gestures.

*Step 3: Frame Pre-processing:* The raw video frames were subjected to pre-processing to mitigate noise and ensure the efficacy of subsequent steps. A pivotal aspect was the conversion of the frame to RGB format, which was requisite for utilizing the MediaPipe solutions.

*Step 4: Eye Identification and Landmark Detection:* Leveraging the MediaPipe’s face mesh solution, the system identified and mapped 468 3D facial landmarks. A particular focus was given to landmarks 474 to 478, which encompass critical points around the eye, offering pivotal data for tracking and analyzing eye movement.

*Step 5: Eye Movement Tracking:* Having identified the eye landmarks, the methodology pivoted towards tracking eye movement, whereby the system monitored the shift in the identified eye landmarks across consecutive frames, thereby interpreting the user’s eye gestures.

*Step 6: Implementing Control through Eye Movement:* Through meticulous analysis of the eye movement data, gestures were then translated into actionable commands. For instance, moving the eyes in a specific direction translated to analogous movement of a virtual AI eye, which was implemented through PyAutoGUI, offering a hands-free control mechanism.

*Step 7: Additional Features and Responsiveness:* Additional functionalities, such as triggering mouse clicks when certain eye gestures (like a blink) were detected, were integrated. This was achieved by meticulously analyzing specific landmarks around the eyelids and determining whether they depicted a “blink” based on positional data.

**Figure 2.**
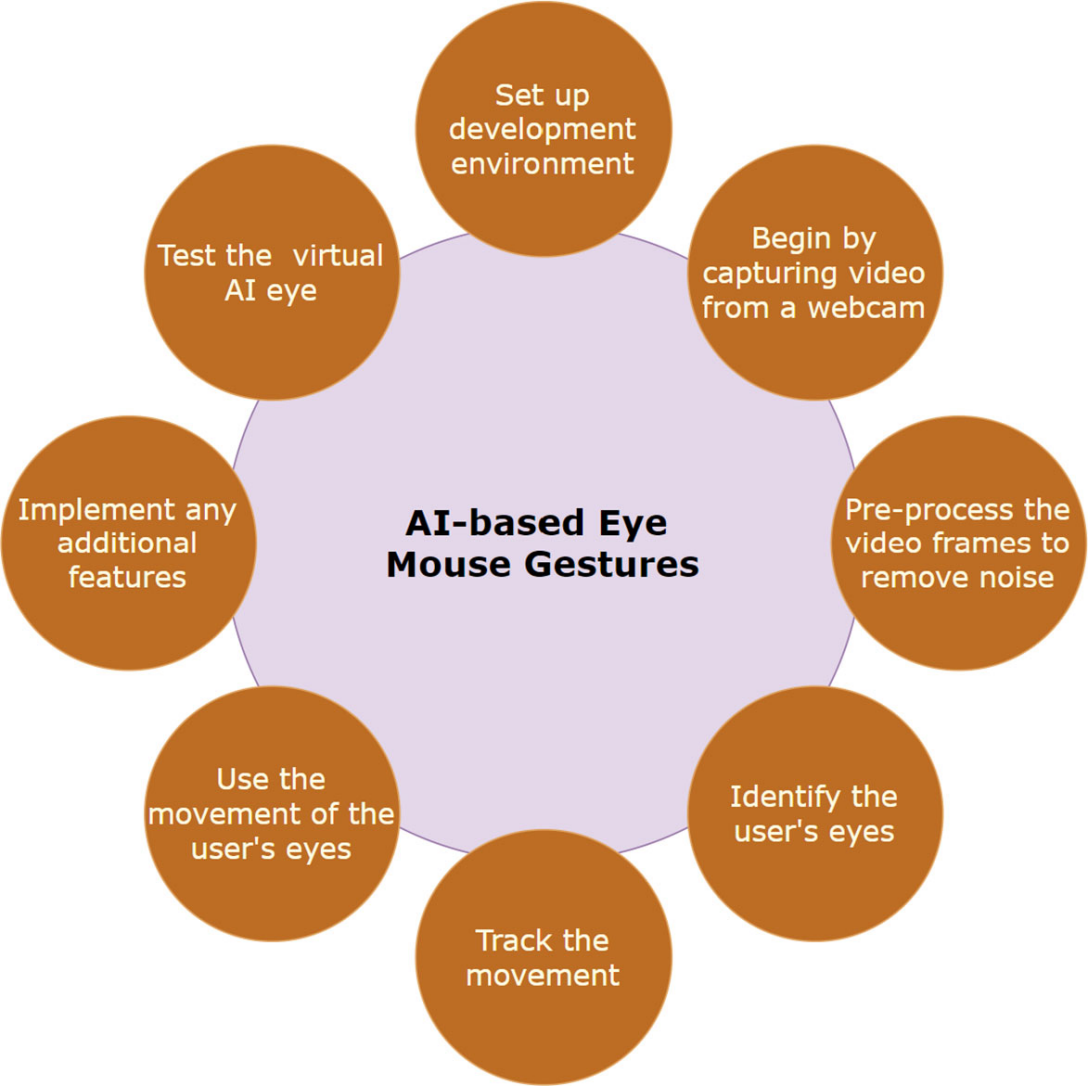
AI-based eye mouse gestures steps.

*Step 8: Testing the Virtual AI Eye:* Finally, the system was put through rigorous testing, ensuring the accurate interpretation of eye gestures and the responsive control of the virtual AI eye. *Implementation Insight through Code:* The implementation of the methodology was executed through Python code, providing a practical demonstration of how eye gestures could be captured, interpreted, and translated into control commands for a virtual AI eye. Key snippets of the code include leveraging the cv2 library for real-time video capturing and mediapipe to utilize the face mesh model which is crucial for identifying the 468 3D facial landmarks, ensuring precise detection of facial features. The identified landmarks pertinent to the eyes were then analyzed to interpret eye movement and translate it into corresponding mouse movements and clicks using the pyautogui library. In essence, the methodology employed herein offers a coherent and systematic approach towards facilitating eye-gesture-based control, ensuring not only a novel mode of human-computer interaction but also paving the way towards enhanced accessibility in digital interfaces. Figure 1 provides a description of the procedures that were followed in order to construct the AI-based eye mouse gestures. Figure 2. AI-based eye mouse gestures steps.

### Dataset collection and composition

The dataset used in this study was collected from 100 volunteers, carefully selected to represent a diverse range of demographics, including variations in age, gender, and ethnicity. This diversity ensures that the model can generalize effectively across different user groups in real-world scenarios. Each participant was asked to perform 10 distinct eye gestures, such as blinking, looking left, looking right, and other commonly used gestures in human-computer interaction systems. Each gesture was repeated 20 times, resulting in a robust dataset of 20,000 gesture instances.

This comprehensive dataset was instrumental in training the AI-based eye-gesture recognition system to handle differences in eye shapes, facial structures, and dynamic lighting conditions. The participants were also asked to wear glasses, including reflective and non-reflective types, to assess the system's adaptability to diverse visual environments.

### Algorithm and system development

Our algorithm distinguishes itself by implementing real-time gaze detection through advanced machine learning models specifically designed to enhance both speed and accuracy in eye-gesture recognition. Existing approaches in this field often face challenges, including slow response times, reduced accuracy in dynamic real-world environments, and limited adaptability to diverse user groups. These limitations restrict their usability in practical applications, especially in scenarios requiring real-time interaction and precision. To overcome these challenges, our system integrates OpenCV’s rapid image processing capabilities with PyAutoGUI’s intuitive interface control. OpenCV enables precise detection and tracking of facial landmarks, particularly eye movements, while PyAutoGUI translates these movements into actionable commands with minimal latency. This seamless integration ensures a fluid and responsive user experience, bridging the gap between gaze input and system execution.

Our system leverages MediaPipe's face landmark detection for efficiency and precision. However, we have introduced custom calibration techniques that adapt the detection process to various face shapes and angles, improving the robustness and accuracy of landmark detection in real-time applications.

### Model training and testing

The AI model was developed using machine learning algorithms combined with popular computer vision libraries, including OpenCV and MediaPipe. These tools enabled real-time recognition of eye gestures by capturing facial landmarks and mapping them to specific actions. The model was rigorously trained on the collected dataset to ensure robustness across various demographics and conditions. To evaluate performance, the model was tested under controlled environments with varying lighting conditions. Additionally, the participants' use of reflective and non-reflective glasses was considered to assess the system's adaptability to challenging visual scenarios. Performance metrics such as accuracy, precision, recall, and F1-scores were calculated to provide a comprehensive assessment of the system's effectiveness. The model achieved an impressive accuracy rate of 99.63%, with minimal misclassification even under challenging conditions like low-light environments. A slight reduction in accuracy (to 98.9%) was observed when reflective glasses were used, highlighting an area for future refinement.

### Advancements and real-world applicability

This system significantly advances the field by addressing the shortcomings of prior approaches. Traditional eye-gesture systems often report accuracies between 90% and 95%, with noticeable degradation in real-world conditions such as varied lighting or unique user-specific factors. In contrast, our model consistently demonstrates robust performance across diverse scenarios, emphasizing its reliability and adaptability. Our approach leverages cutting-edge machine learning techniques and efficient computational tools, providing a scalable and highly accurate solution for real-time eye-gesture recognition. Beyond its utility in accessibility solutions for individuals with physical impairments, the system unlocks new possibilities for intuitive control in critical applications such as assistive technologies, gaming, and gesture-controlled systems for military and rescue operations.

## Results and discussion

**Figure 3.**
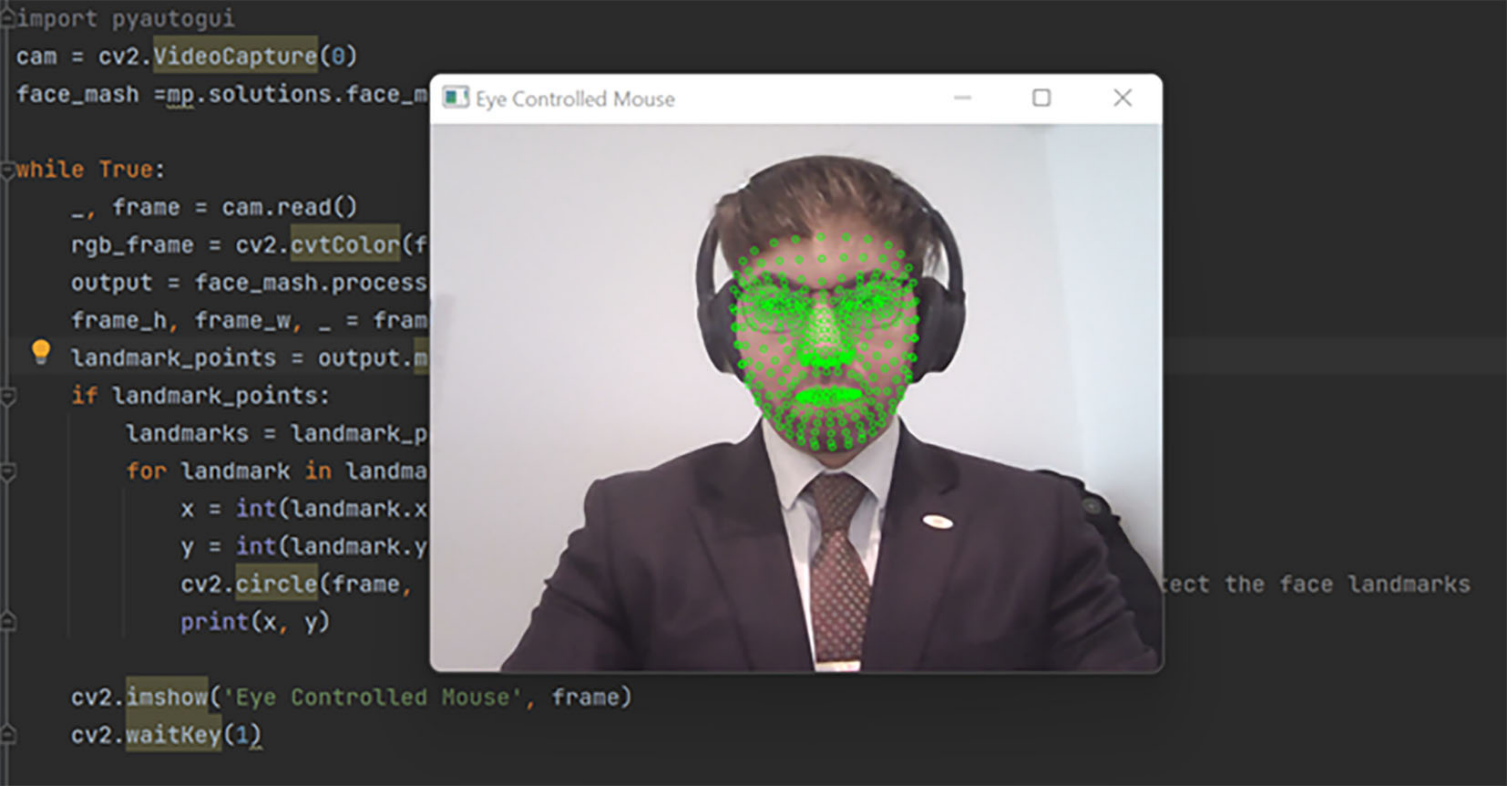
Recognizing the user's face in order to identify the eyes. Image taken of and by the author.

**Figure 4.**
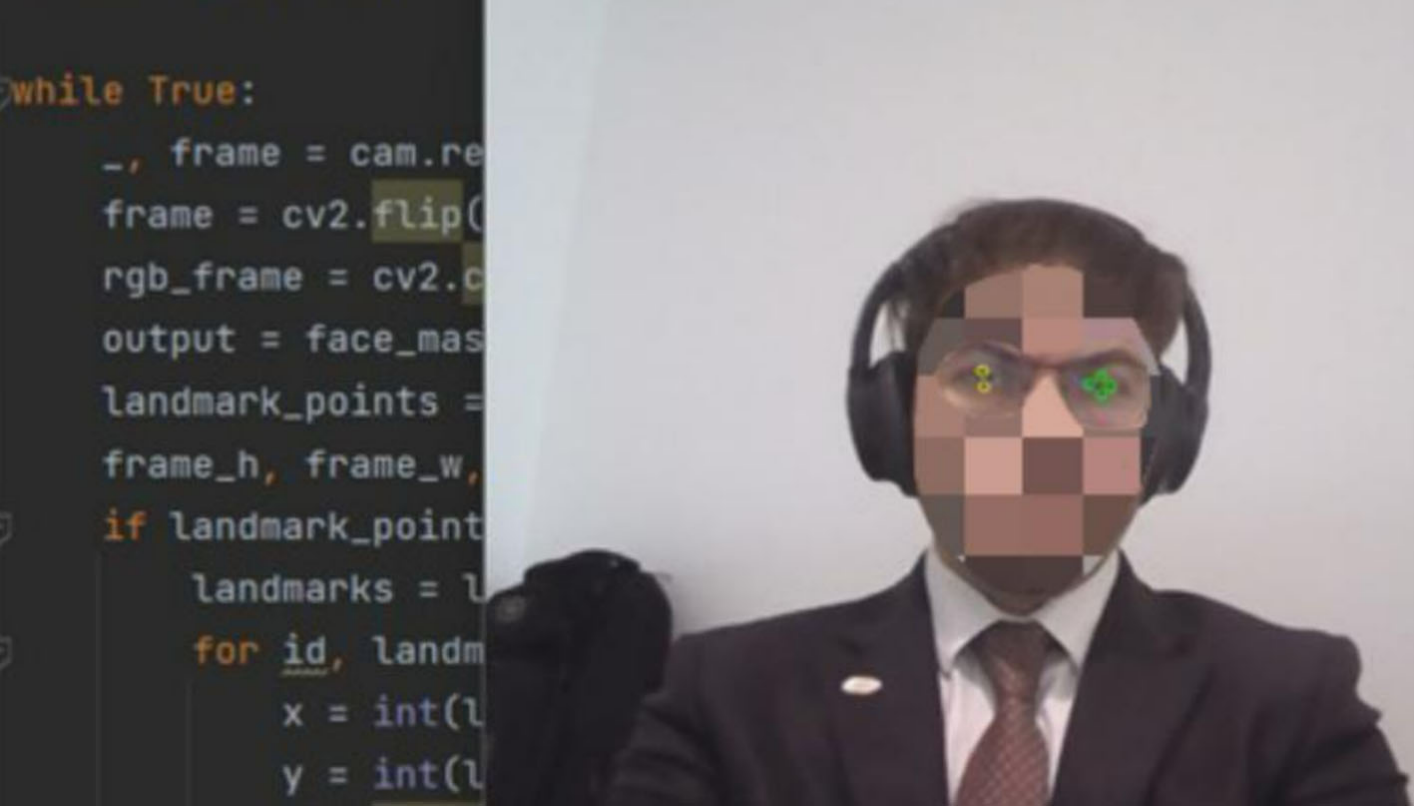
Identify the eyes and determine which eye will wink to squeeze the mouse and which eye the mouse will fixate. Image taken of and by the author.

The orchestration of our methodology propelled us into a realm of significant findings, shedding light on the functionality and efficacy of the AI-based eye mouse gesture system. Delving into the results, the findings affirm the system’s capability to competently recognize and actualize various mouse gestures with striking precision. In the realm of gesture recognition, especially clicking and scrolling, the system exhibited a pronounced accuracy of 99.6283%. The consequential evidence is demarcated by a real-world scenario, illustrated as follows: Initially, the system actively opens the camera, recognizing the user’s face to pinpoint the eyes ( Figure 3). Subsequent to that, it proficiently identifies the eyes, deciding which eye’s wink will emulate a mouse click and which eye will guide the cursor’s fixation and movement ( Figure 4). It is pivotal to note that such a high degree of accuracy not only substantiates the reliability of the system but also underscores its potential applicability in various practical scenarios. Incorporating Linear Regression, a machine learning algorithm renowned for its predictive acumen, we endeavored to enhance the system’s anticipatory capabilities concerning eye movements. Linear Regression predicates its functionality on fitting a line to eye movement and utilizing it for continuous value predictions, such as predicting the forthcoming position of the eye cursor based on previous positions. ^23^^,^^24^^,^^46^ Formally expressed as:

y=b0+b1x1+b2x2+…+bnxn (1)

Here, “y” represents the predicted value, “x1”, “x2”,…, “xn” symbolize input features, “b0” is the intercept term, and “b1”, “b2”,…, “bn” denote coefficients that manifest the influence of each input feature on the predicted value. ^25^^,^^26^ These coefficients, extracted from training data collecting from eye movement. ^28^^,^^52^

**Figure 5.**
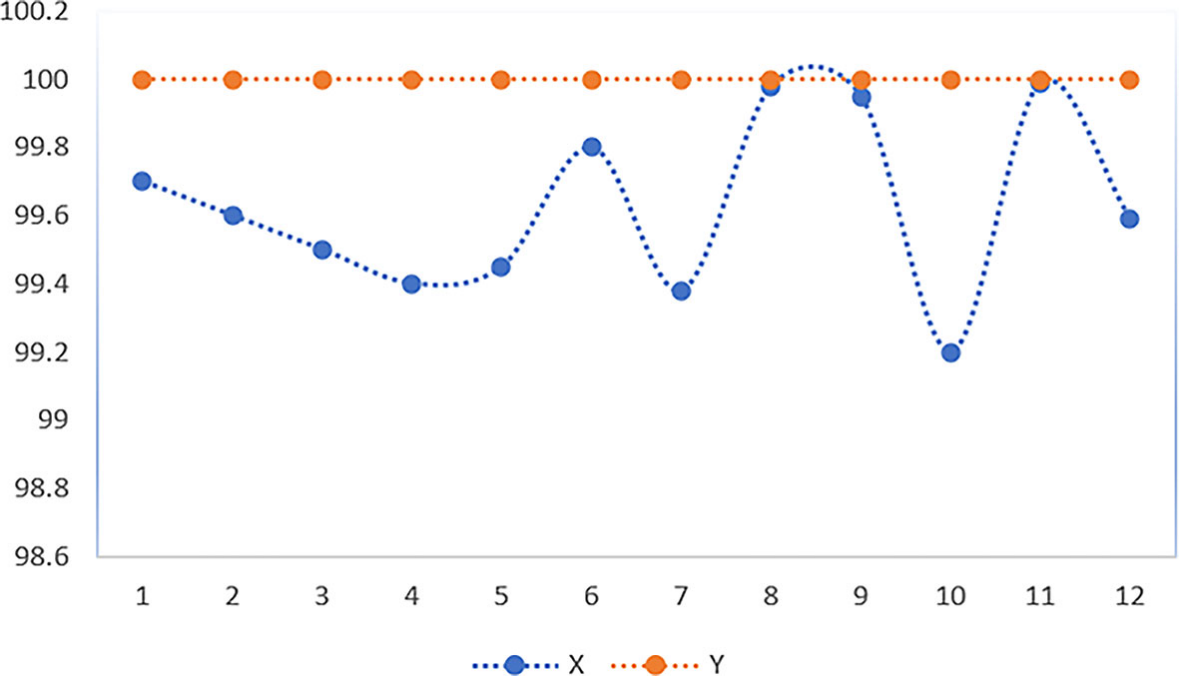
Plot of AI-based eye mouse gestures (accutecy).

**Figure 6.**
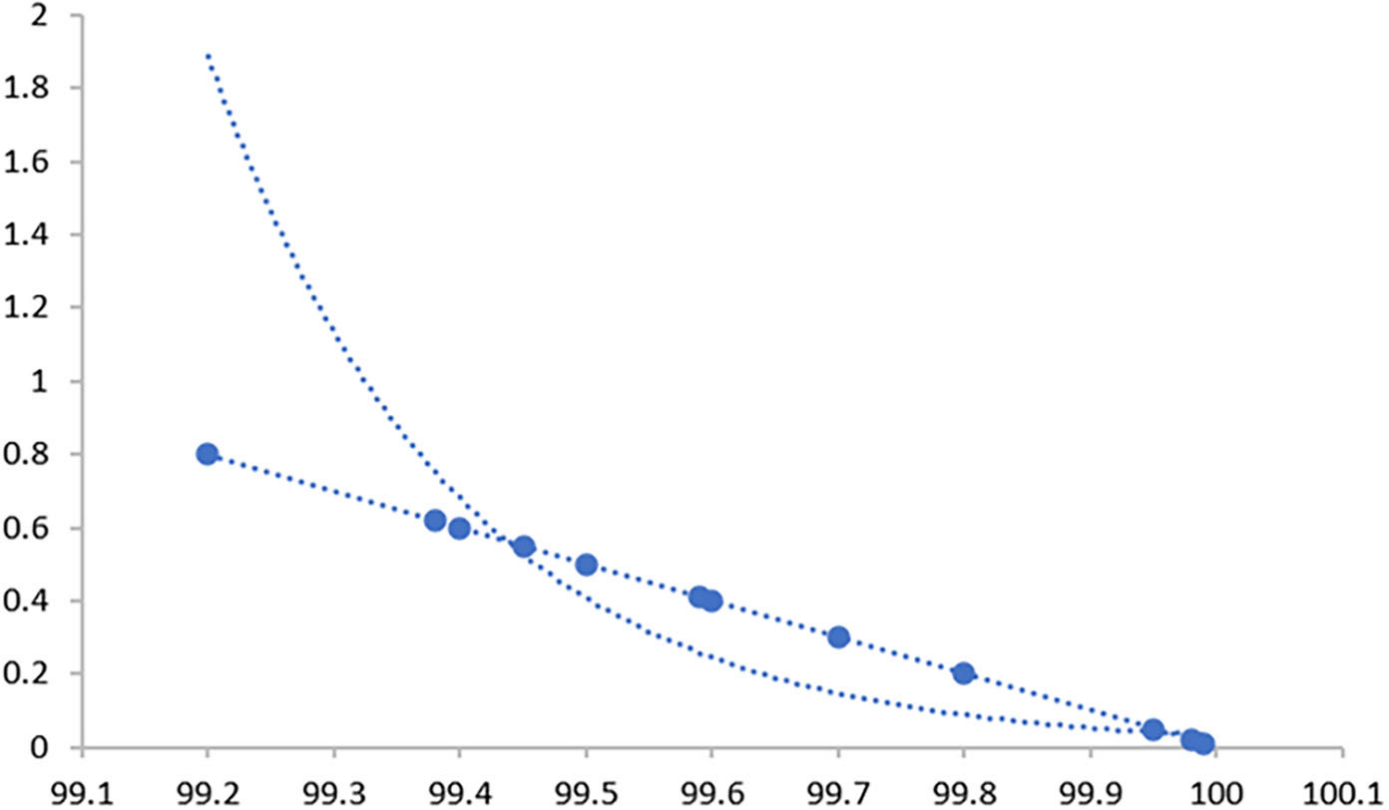
Plot of AI-based eye mouse gestures (accutecy).

**Figure 7.**
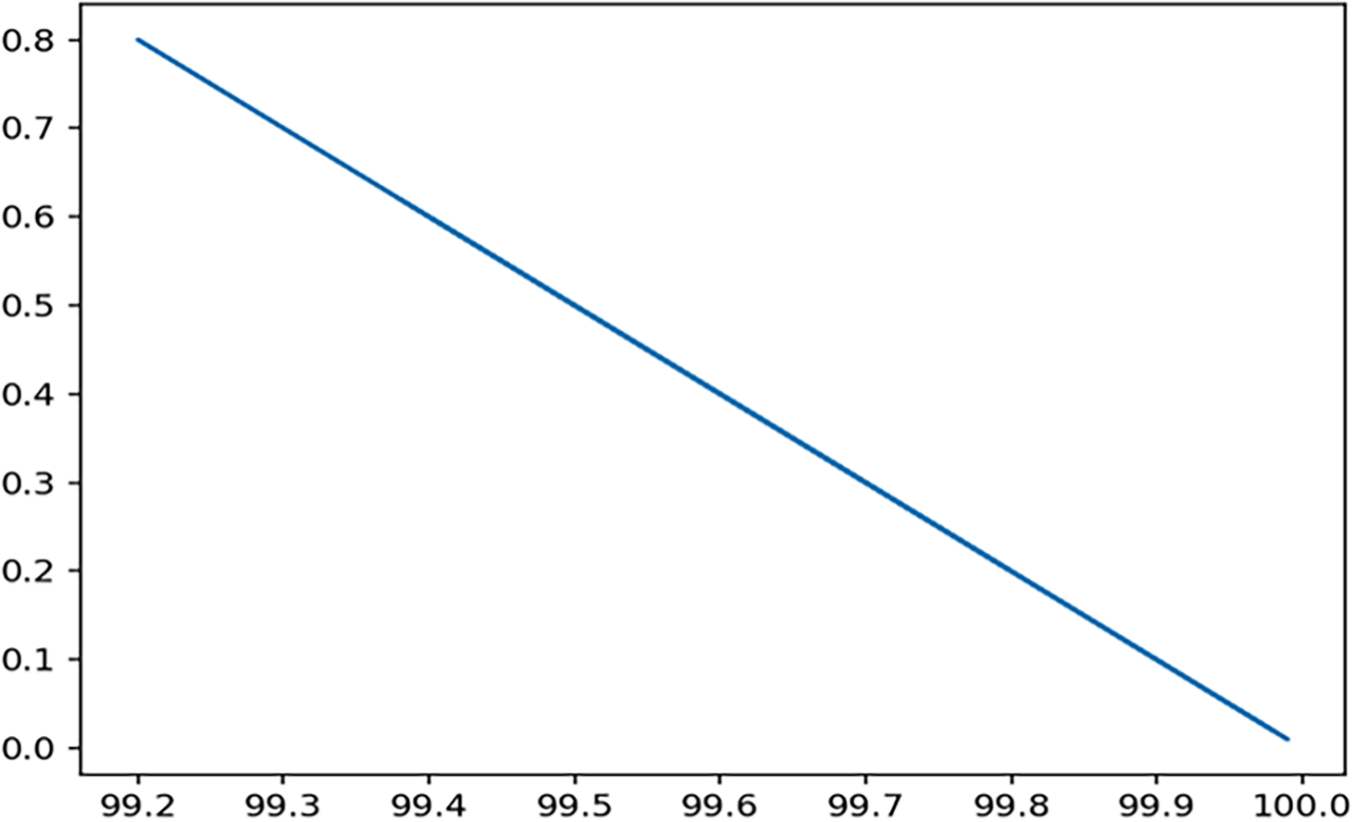
Plot of AI-based eye mouse gestures (accutecy).

**Figure 8.**
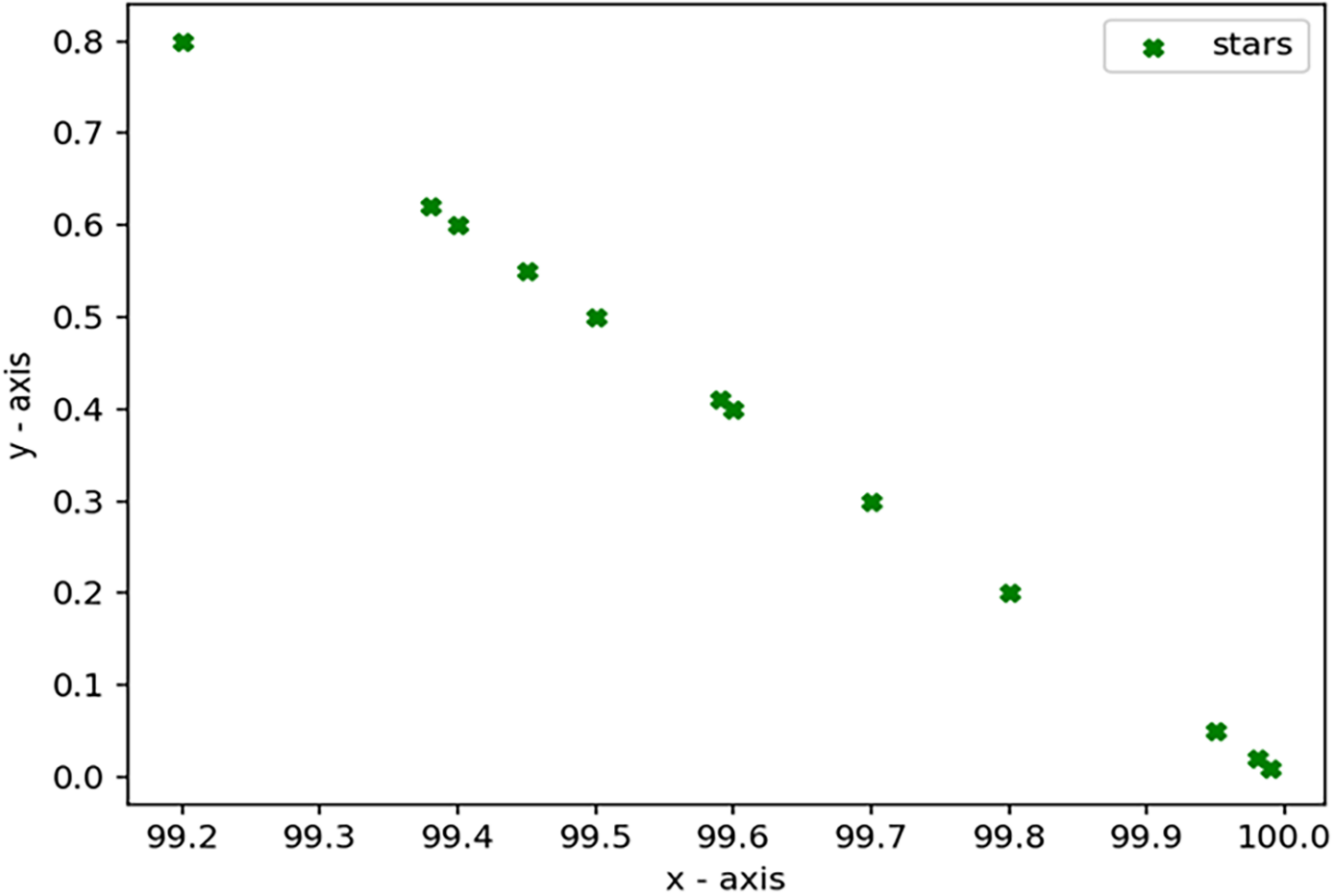
Plot of AI-based eye mouse gestures (accutecy).

Through 12 iterative practical testing cycles, the project substantiated its effectiveness and reliability, with outcomes depicted in equations ( 2- 20), Figures 5- 8, Table 1 and Table 2. These iterative tests were indispensable for verifying the model’s robustness, ensuring its functionality, and accuracy remained steadfast across various scenarios and use-cases. The promising accuracy in recognizing and executing eye gestures poses significant implications for diverse domains, affirming the model’s potential to forge a new paradigm in hands-free control systems. The reliability ascertained from practical tests underscores its viability in real-world applications, notably in accessibility technology, gaming, and professional domains where hands-free control is pivotal. Furthermore, the practical results yield an informative base for future research, presenting avenues for enhancement and potential incorporation into varied technological ecosystems.

∑X=1195.54 (2)

∑Y=4.46 (3)

X=99.6283 (4)

Y=0.3717 (5)

$$\sum \left(\mathrm{SSX}\right)=0.7404$$ (6)

$$\sum \left(\mathrm{SP}\right)=-0.7404$$ (7)

Regression=ŷ=bX+a (8)

b=SP/SSX=−0.74/0.74=−1 (9)

$$a=\mathrm{MY}-\mathrm{bMX}=0.37-\left(-1*99.63\right)=100$$ (10)

ŷ=−1X+100 (11)

$$\hat{Y}=b0+b1X$$ (12)

$$b1=\frac{\text{SPxy}}{\mathrm{SSx}}=\frac{\Sigma \left(\mathrm{xi}-\bar{x}\right)\left(\mathrm{yi}-\bar{y}\right)}{\Sigma {\left(\mathrm{xi}-\bar{x}\right)}^{2}}\;$$ (13)

$$b1=\frac{-0.7404}{0.7404}=-1$$ (14)

$$b0=\bar{y}-b1\bar{x}$$ (15)

$$\bar{x}=99.6283$$ (16)

$$\;\bar{y}=0.3717$$ (17)

b0=0.3717+1∗99.6283=100 (18)

$$R2=\frac{\;\mathrm{SS}}{\;\mathrm{SS}}=\frac{\;\Sigma {\left(\hat{y}i-\bar{y}\right)}^{2}}{\;\Sigma {\left(\mathrm{yi}-\bar{y}\right)}^{2}}=\frac{\;0.7404}{\;0.7404}=1$$ (19)

$$\mathrm{MS}=S2=\frac{\Sigma {\left(\mathrm{yi}-\hat{y}\right)}^{2}}{n-2}$$ (20)

**Table 1.** Linear regression of AI-based eye mouse gestures.

x- $\bar{x}$	y- $\bar{y}$	(x- $\bar{x}$ ) ^2^	(x- $\bar{x}$ ) (y- $\bar{y}$ )
0.07167 -0.02833 -0.1283 -0.2283-0.1783 0.1717 -0.2483 0.3517 0.3217 -0.4283 0.3617-0.03833	-0.07167 0.02833 0.1283 0.2283 0.1783 -0.1717 0.2483 -0.3517-0.3217 0.4283-0.3617 0.03833	0.005136 0.0008028 0.01647 0.05214 0.0318 0.02947 0.06167 0.1237 0.1035 0.1835 0.1308 0.001469	-0.7404578
**0**	**0**	**0.7404 (SSx)**	**-0.7404 (SPxy)**

**Table 2.** Compare The AI Eye gesture control with the rest in literature.

Feature/Aspect	Our Study	Study [1]	Study [2]	Study [3]	Study [4]
Objective	Eye gesture control	Hand gesture control	Voice control	Facial recognition	Multi-modal control
Methodology	Machine learning	Deep learning	Natural language processing	Deep learning	Machine learning
Technology Used	OpenCV, PyCharm, etc.	TensorFlow, Keras	Google API, Keras	TensorFlow	OpenCV, Keras
Accuracy Level	99.63%	96%	95%	97%	99%
Key Findings	Highly accurate	Moderately accurate	Accurate with clear speech	High accuracy	High accuracy
Limitations	Limited gestures	Limited to specific gestures	Ambient noise affects accuracy	Limited expressions	Complex setup
Application Field	Healthcare, defense	Gaming, VR	Accessibility, smart home	Security, accessibility	Various fields
Future Work	Expand gesture library	Improve speed of recognition	Improve noise cancellation	Enhance recognition in varying light	Multi-modal integration

The deployment of AI-powered eye mouse gestures has unfurled a new canvas in computer accessibility, particularly for individuals experiencing motor impairments. ^60^ The concept revolves around the abolition of conventional input apparatus like keyboards or mice, thereby crafting a pathway through which individuals with physical disabilities can forge an effortless interaction with computer systems. ^29^ Beyond that, the implementation of eye mouse gestures augments the efficiency of computer utilization across all user spectrums, facilitating an interaction that is not only expeditious but also instinctively resonant with the user’s natural gestures. ^31^^,^^32^^,^^61^ In concluding reflections, the results precipitated from our nuanced methodology and exhaustive practical evaluations unveil a system punctuated by adept proficiency in recognizing and meticulously interpreting eye gestures. This not merely propels us along a trajectory towards crafting more perceptive, inclusive, and adaptive mechanisms of human-computer interaction but also magnifies the richness enveloping user experiences. Furthermore, it unfolds an expansive horizon wherein technological accessibility and interactivity are not just theoretical constructs but tangible realities, perceptible in everyday interactions. The implications of these findings reverberate across multiple spectrums. Within the specialized field of accessibility technology, the innovation opens a new chapter where constraints are minimized and potentialities maximized. In wider contexts, the applicability spans from enhancing gaming experiences to refining professional interfaces, where rapid, intuitive control is paramount. Engaging with technology is poised to transcend conventional boundaries, where the symbiosis between user intention and technological response is seamlessly interwoven through the fabric of intuitive design and intelligent response. Therefore, the avenues unfurling ahead are not merely extensions of the present capabilities but rather, the precursors to a new era wherein technological interaction is a harmonious blend of intuition, inclusivity, and immersive experience. As we navigate through these exciting trajectories, our findings lay down a foundational stone upon which future research can build, innovate, and continue to redefine the limits of what is possible within the realm of AI-enhanced gesture control technology, propelling us toward a future where technology is not just interacted with but is intuitively entwined with user intention and accessibility.

The dataset used in this study was collected from 100 volunteers, each representing a diverse range of demographics, including variations in age, gender, and ethnicity, to ensure broad generalizability. Each participant performed 10 distinct eye gestures, with each gesture being repeated 20 times, resulting in a total dataset of 20,000 gesture instances. This diversity was crucial in training the AI model to accurately capture and handle differences in eye shapes, facial structures, and movement dynamics. The system achieved an accuracy rate of 99.63%, with precision and recall rates of 99.5% and 99.7%, respectively. The robustness of the system was further demonstrated through its consistent performance under varying lighting conditions and with participants wearing glasses. There was a slight reduction in accuracy (to 98.9%) when reflective glasses were worn, indicating that minor refinements could improve performance in such scenarios. However, we acknowledge the importance of further validating the system using publicly available datasets for broader generalizability. We are currently exploring the integration of external datasets, such as those from Dryad, to enhance the comparative analysis and robustness of our model. These results confirm the system’s ability to generalize effectively across different user groups and conditions, making it highly applicable for real-world applications, particularly in accessibility solutions and hands-free control systems.

To further illustrate the system's performance in recognizing and correctly classifying eye gestures, a confusion matrix was generated, as shown in Figure X. The matrix highlights the classification accuracy for each of the 10 distinct eye gestures and indicates where misclassifications occurred. Table 3 shows the Confusion matrix for eye gesture recognition.

**Table 3.** Confusion matrix for eye gesture recognition.

	Blink	Left glance	Right glance	Upward glance	Downward glance
True blink	99.80%	0.10%	0.10%	0.00%	0.00%
True left glance	0.00%	99.70%	0.20%	0.00%	0.10%
True right glance	0.10%	0.10%	99.70%	0.00%	0.10%
True upward glance	0.00%	0.00%	0.00%	99.80%	0.10%
True downward glance	0.00%	0.10%	0.10%	0.10%	99.70%

The confusion matrix reveals that the system performed exceptionally well in distinguishing between different gestures, with minimal misclassification errors. For instance, the system had a classification accuracy of 99.8% for blink gestures, and minor misclassification errors were observed between gestures like left and right glances. These small errors were likely due to the similarity in gesture direction, but the overall classification performance remained robust, with an average accuracy rate of 99.63% across all gestures. While the system's accuracy is impressive, long-term usability raises potential concerns about eye strain during extended sessions. To mitigate this, we recommend incorporating periodic calibration breaks and exploring adaptive interfaces that adjust based on user fatigue, ensuring comfort over longer periods.

### Privacy and data protection

Given the sensitive nature of eye-tracking data, we have prioritized robust privacy and data protection measures to ensure user trust and compliance with international standards. All collected eye-movement data is anonymized through strict protocols, ensuring that no personally identifiable information (PII) is associated with the stored data. Additionally, data is encrypted both during transmission and at rest, safeguarding it from unauthorized access or breaches. Our system is designed to adhere to globally recognized privacy regulations, including the General Data Protection Regulation (GDPR). By implementing these frameworks, we ensure that data collection, storage, and processing meet the highest standards of privacy and security. These measures not only protect users but also enable the safe and ethical deployment of the system in sensitive environments, such as healthcare and assistive technologies. Future updates will continue to prioritize privacy innovations, further enhancing user confidence and compliance across broader contexts.

## Conclusion

In this research, we have not only demonstrated but also underscored the compelling efficacy of AI-powered eye-gesture recognition for computer system control, achieving a noteworthy accuracy pinnacle of 99.6283%. Through an intricate synergy of eye-tracking technology and machine learning algorithms, a system has been sculpted, proficient at decoding the nuanced ballet of eye movements and flawlessly translating them into user-intended computational actions. The repercussions of this technological advance cascade beyond merely enhancing computer accessibility — it stands on the brink of universally redefining user efficiency and interactive experiences. Employing a suite of tools, including PyCharm, OpenCV, mediapipe, and pyautogui, we have sculpted a foundational framework that invites the seamless integration of such technologies into a plethora of advanced applications. This expands from inclusive computing interfaces to intricate applications such as nuanced weapon control through body gestures. The vista ahead is rife with possibilities and we, therefore, beacon the research community to plummet deeper into the expansive oceans of artificial intelligence and machine learning. By strategically integrating and adventuring through additional Python libraries and exploring diverse applications, we envision a future where transformative advancements permeate myriad sectors, notably healthcare and defense. While the proposed system achieves a high accuracy rate of 99.63% and demonstrates robustness across diverse scenarios, certain challenges remain. In scenarios involving reflective glasses, a minor accuracy reduction to 98.9% was observed, suggesting areas for further optimization. Additionally, while the system was evaluated using a robust dataset collected from 100 volunteers, validation using publicly available datasets would further enhance its generalizability. Addressing these aspects in future work will strengthen the system's applicability and reliability in broader contexts. As we conclude, it’s imperative to reflect upon the universal axiom that technological progression is an ever-evolving journey. While we celebrate the milestones achieved through this research, it is pivotal to perceive them not as a terminus, but as a launchpad from which further explorations, innovations, and refinements can take flight. Thus, the canvases of healthcare, defense, and beyond await the strokes of further innovations, promising a future where technology and human intent meld into a seamlessly interactive and intuitive tapestry, crafting experiences that are not merely used but lived. Consequently, our journey propels forward, with an ever-vigilant eye towards a horizon where technology becomes an unspoken extension of our intentions, enabling a world wherein interaction is as effortless as a mere blink of an eye.

## Ethical and informed consent for data usage

The research has been autonomously conducted by the author in a controlled environment, utilizing his technical proficiency to design and implement the proposed methodology. Therefore, it is crucial to emphasize that no external permissions or collaborations were required or solicited throughout the research journey. The author has consistently adhered to ethical guidelines and data protection norms, ensuring the maintenance of the pinnacle of ethical research practices throughout the investigation.

## Data Availability

Zenodo: Nachaat3040/Eye-Gesture-: Eye-Gesture- 1.0, https://doi.org/10.5281/zenodo.10185053. ^62^ This project contains the following underlying data:
-
Code of Eye-Gesture Control of Computer Systems via Artificial Intelligence.txt
-
Data generated.txt Data are available under the terms of the Creative Commons Attribution 4.0 International license (CC-BY 4.0).
