# Supplementary material for: Eye-gesture control of computer systems via artificial intelligence
Source: F1000Res. 2025 Feb 25;13:109. Originally published 2024 Feb 19. [Version 3] doi: 10.12688/f1000research.144962.3 (PMC11876798; doi:10.12688/f1000research.144962.3)
Supplement: Peer review history

# Reviewer report: Reviewer response for version 3

**DOI:** 10.5256/f1000research.178296.r368421

**Published:** 2025-03-03

**Authors:** Gaurav Meena

**Affiliations:** 1 Central University of Rajasthan, Ajmer, India

Accepted. The authors incorporated all the changes.

Is the work clearly and accurately presented and does it cite the current literature?

Partly

If applicable, is the statistical analysis and its interpretation appropriate?

Not applicable

Are all the source data underlying the results available to ensure full reproducibility?

Partly

Is the study design appropriate and is the work technically sound?

Partly

Are the conclusions drawn adequately supported by the results?

Partly

Are sufficient details of methods and analysis provided to allow replication by others?

Partly

Reviewer Expertise:

Deep Learning, Artificial Intelligence

I confirm that I have read this submission and believe that I have an appropriate level of expertise to confirm that it is of an acceptable scientific standard.

# Reviewer report: Reviewer response for version 2

**DOI:** 10.5256/f1000research.175922.r360627

**Published:** 2025-01-24

**Authors:** Gaurav Meena

**Affiliations:** 1 Central University of Rajasthan, Ajmer, India

The manuscript presents several significant issues that need to be addressed before it can be considered for indexing. Please find my detailed comments and concerns below:

 1. Please add two paragraphs in the introduction: a) objectives and motivations tied to gaps in the literature; b) research questions.

 2. The literature review of the article doesn't provide comprehensive coverage of related work. Most troublesome is the significant gap in coverage of the state-of-the-art. A few important pieces of literature about deep learning are not cited, for example.

 (a). FSTL-SA: few-shot transfer learning for sentiment analysis from facial expressions, Meena G .et.al., 2024 (Ref 1)

 3. It is not clear how the authors used the embedding techniques. Please provide more explanations.

 4. It is not clear how the authors fine-tuned the model. Please add experiments and details.

 5. It is not clear how the parameters of the models are compared.

 6. I would strongly advise including all major works in 2023 and 2024 and drawing a tabular comparison among your work and other works. A few works worth comparing, referring to, or citing are below:

 (b).  Monkeypox recognition and prediction from visuals using deep transfer learning-based neural networks, Meena G .et.al., 2024 (Ref 2) 

 7. There is no such sub-section mentioning the details about the dataset size. I couldn’t find it anywhere regarding the size or quantity of the dataset.

 8. I'd recommend adding some possible improvements to the proposed approach.

 9. What are the avenues for future research or improvements identified based on this study?

 10. The challenges in the work need to be stated (As mentioned in the Title)

 11. What are the challenges in the dataset?

Is the work clearly and accurately presented and does it cite the current literature?

Partly

If applicable, is the statistical analysis and its interpretation appropriate?

Not applicable

Are all the source data underlying the results available to ensure full reproducibility?

Partly

Is the study design appropriate and is the work technically sound?

Partly

Are the conclusions drawn adequately supported by the results?

Partly

Are sufficient details of methods and analysis provided to allow replication by others?

Partly

Reviewer Expertise:

Deep Learning, Artificial Intelligence

I confirm that I have read this submission and believe that I have an appropriate level of expertise to confirm that it is of an acceptable scientific standard, however I have significant reservations, as outlined above.

## References

[1] : FSTL-SA: few-shot transfer learning for sentiment analysis from facial expressions. *Multimedia Tools and Applications* .2024; 10.1007/s11042-024-20518-y 10.1007/s11042-024-20518-y

[2] : Monkeypox recognition and prediction from visuals using deep transfer learning-based neural networks. *Multimedia Tools and Applications* .2024;83(28) : 10.1007/s11042-024-18437-z 71695-71719 10.1007/s11042-024-18437-z

# Response

**Published:** 2025-02-14

**Authors:** Nachaat Mohamed

**Affiliations:** HLS, Rabdan Academy, Abu Dhabi, Abu Dhabi, United Arab Emirates

Dear Dr. Meena,

 We sincerely appreciate your thorough review and valuable feedback on our manuscript. Your insights have been instrumental in enhancing the quality and clarity of our work. Below, we address each of your comments in detail:

**1. Introduction Enhancements:**

- **Objectives and Motivations:** We have expanded the introduction to include a detailed discussion of the objectives and motivations of our study, explicitly linking them to identified gaps in the existing literature.
- **Research Questions:** A new subsection outlining the specific research questions guiding our investigation has been added to provide clarity on the study's focus.

**2. Comprehensive Literature Review:**

- We have conducted an extensive review of recent literature, particularly focusing on state-of-the-art developments in deep learning. Notably, we have included the following reference to enrich our discussion:
  - Meena, G., et al. (2024). "FSTL-SA: Few-Shot Transfer Learning for Sentiment Analysis from Facial Expressions."

**3. Embedding Techniques Explanation:**

- We have elaborated on the embedding techniques utilized in our study, providing a comprehensive explanation of their implementation and role within our methodology.

**4. Model Fine-Tuning Details:**

- Detailed descriptions of the model fine-tuning process have been incorporated, including the specific experiments conducted and the parameters adjusted to optimize performance.

**5. Model Parameter Comparison:**

- We have clarified the comparative analysis of model parameters, outlining the criteria and metrics used to evaluate and contrast the performance of different models.

**6. Inclusion of Recent Works and Comparative Analysis:**

- Our literature review now encompasses major works from 2023 and 2024. We have also included a comparative table that juxtaposes our findings with those of recent studies, including:
  - Meena, G., et al. (2024). "Monkeypox Recognition and Prediction from Visuals Using Deep Transfer Learning-Based Neural Networks."

**7. Dataset Size Specification:**

- A subsection titled "Dataset Description" has been added to the Methodology section, detailing the size and composition of the dataset used in our study.

**8. Proposed Approach Improvements:**

- We have included a discussion on potential improvements to our proposed approach, highlighting areas for enhancement and refinement.

**9. Future Research Directions:**

- A "Future Work" subsection has been added to outline avenues for future research, building upon the findings of our current study.

**10. Challenges in the Work:**

- We have articulated the challenges encountered during our research, providing a candid discussion of the obstacles faced and how they were addressed.

**11. Dataset Challenges:**

- The "Dataset Description" subsection also includes an analysis of the challenges inherent in our dataset, discussing limitations and potential impacts on our findings.

We trust that these revisions comprehensively address your concerns. Your constructive feedback has been invaluable in strengthening our manuscript, and we are grateful for your thoughtful suggestions.

 Warm regards,

 Dr. Nachaat Mohamed

# Reviewer report: Reviewer response for version 2

**DOI:** 10.5256/f1000research.175922.r350253

**Published:** 2024-12-24

**Authors:** Haodong Chen

**Affiliations:** 1 School of Engineering, University of Maryland at College Park, College Park, Maryland, USA

1. Authors should refer to 3-4 related papers to strengthen the review of existing literature and highlight the novelty of this work Suggested papers include:

**a. Real-time human-computer interaction using eye gazes. DOI: 10.1016/j.mfglet.2023.07.024**

 This paper explores a real-time system that uses eye gazes for interacting with computers, which is very relevant to the authors' focus on eye-gesture recognition. By citing this work, the authors can highlight how their AI-driven approach improves accuracy and responsiveness compared to existing gaze-based systems.

**b. Real-Time Multi-Modal Human–Robot Collaboration Using Gestures and Speech. DOI: 10.1115/1.4054297**

 This study looks at combining gestures and speech for better collaboration between humans and robots. Referencing it will help the authors position their eye-gesture control system within the broader field of multi-modal interactions and emphasize the unique advantage of focusing solely on eye movements.

**c. Fine-grained activity classification in assembly based on multi-visual modalities. DOI: 10.1007/s10845-023-02152-x**

 This research dives into detailed activity classification using multiple visual inputs, demonstrating the effectiveness of using diverse visual data. Citing this paper allows the authors to showcase the precision and targeted application of their eye-gesture recognition system, contrasting it with broader multi-visual activity classification methods.

 2. Have the authors considered discussing the potential issue of eye strain or fatigue during long-term use of the eye-gesture system? Could they propose possible mitigation strategies, such as adaptive interfaces or periodic calibration breaks?

 3. Can the authors provide more detail on the diversity of the dataset, such as specific demographic categories (e.g., age, gender, ethnicity), and explain how this diversity impacts the system's performance across different user groups?

Is the work clearly and accurately presented and does it cite the current literature?

Yes

If applicable, is the statistical analysis and its interpretation appropriate?

Partly

Are all the source data underlying the results available to ensure full reproducibility?

Partly

Is the study design appropriate and is the work technically sound?

Partly

Are the conclusions drawn adequately supported by the results?

Partly

Are sufficient details of methods and analysis provided to allow replication by others?

Partly

Reviewer Expertise:

Computer Vision, Artificial Intelligence, AI, Machine Learning, Deep Learning, Human-Computer Interaction (HCI), Eye Gaze Estimation, Gesture Recognition, Industrial AI, generative AI

I confirm that I have read this submission and believe that I have an appropriate level of expertise to confirm that it is of an acceptable scientific standard, however I have significant reservations, as outlined above.

## References

[1] : Real-Time Multi-Modal Human–Robot Collaboration Using Gestures and Speech. *Journal of Manufacturing Science and Engineering* .2022;144(10) : 10.1115/1.4054297 10.1115/1.4054297

[2] : Real-time human-computer interaction using eye gazes. *Manufacturing Letters* .2023;35: 10.1016/j.mfglet.2023.07.024 883-894 10.1016/j.mfglet.2023.07.024

[3] : Fine-grained activity classification in assembly based on multi-visual modalities. *Journal of Intelligent Manufacturing* .2024;35(5) : 10.1007/s10845-023-02152-x 2215-2233 10.1007/s10845-023-02152-x

# Response

**Published:** 2025-02-14

**Authors:** Nachaat Mohamed

**Affiliations:** HLS, Rabdan Academy, Abu Dhabi, Abu Dhabi, United Arab Emirates

**Dear Prof. Dr. Haodong Chen,**

 We sincerely appreciate your valuable feedback and constructive suggestions, which have helped us enhance the clarity, depth, and rigor of our manuscript. Below, we provide detailed responses to each of your comments and outline the specific improvements made.

**1. Strengthening the Review of Existing Literature**

 We have revised the **Related Work** section to incorporate references to the suggested papers. Specifically:

- We cited [64] to compare our system with gaze-based human-computer interaction approaches, emphasizing how our method improves accuracy and responsiveness.
- Reference [63] has been added to position our work within the broader field of multi-modal interactions, differentiating our approach as a **purely eye-gesture-based** system.
- We also included [65] to contrast our system with **multi-visual classification** approaches, highlighting our lightweight and real-time solution.

These additions strengthen the theoretical foundation of our study and better contextualize its contributions.

**2. Addressing Eye Strain and Fatigue in Long-Term Use**

  A new subsection titled **User Comfort and Long-Term Usability** has been added to the **Discussion** section. This section acknowledges that prolonged use of eye-tracking systems may lead to discomfort or fatigue. To mitigate this, we propose:

- **Adaptive Sensitivity Adjustments** to dynamically modify gesture responsiveness based on detected user fatigue.
- **Periodic Calibration Breaks** to prompt users to rest at optimal intervals.
- **Customizable Interaction Modes** to allow users to personalize gesture sensitivity according to their comfort levels.

These refinements ensure that our system remains **comfortable and user-friendly**, even during extended usage periods.

**3. Expanding Dataset Diversity Details**

 The **Dataset Collection and Composition** subsection in the **Methodology** section has been expanded to include:

- Detailed demographic breakdowns of **age groups (young adults to seniors), gender (male and female), and ethnic backgrounds** to ensure system generalizability.
- An analysis of how this diversity impacts system performance, confirming **no significant bias across user groups** and maintaining **high accuracy across all demographics**.
- A discussion on how our model was tested under **varied visual conditions**, including participants wearing both reflective and non-reflective glasses.

This ensures that our system is robust, inclusive, and applicable to **real-world, diverse user bases**.

**Conclusion**

 We appreciate your insightful feedback, which has allowed us to significantly enhance our manuscript. The requested revisions have been implemented, and we believe the updated version now provides a **more comprehensive review of existing literature, a stronger dataset description, and a well-justified discussion on long-term usability considerations**.

 Thank you again for your time and thoughtful comments. We look forward to your feedback on the revised manuscript.

 Best regards,

 Dr. Nachaat Mohamed

# Reviewer report: Reviewer response for version 1

**DOI:** 10.5256/f1000research.158833.r285544

**Published:** 2024-09-03

**Authors:** Haodong Chen

**Affiliations:** 1 School of Engineering, University of Maryland at College Park, College Park, Maryland, USA

**Summary of the Article:**

 The paper introduces an advanced AI-based system that utilizes eye gestures to control computer systems, achieving an impressive accuracy of over 99.63%. This system leverages widely used libraries such as OpenCV, mediapipe, and pyautogui, making it accessible for implementation and demonstrating significant potential in applications ranging from accessibility enhancements for individuals with physical impairments to intuitive interaction in various fields, including military and rescue operations.

**Major Points:**

1. **Long-Term Usability and Comfort:** The proposed eye gaze system presents a highly accurate and innovative approach. However, one of the key aspects that the paper does not address is the long-term usability and comfort of such eye gesture systems. Prolonged use could lead to eye strain or fatigue, which may diminish the system's practicality over time. Including an analysis or discussion on this aspect would strengthen the paper significantly.
2. **Privacy Concerns:** Another major consideration is the potential privacy implications associated with continuous eye tracking. The paper does not discuss how the system handles or safeguards the sensitive data it collects from users. Addressing these concerns is crucial, especially for systems that may be deployed in sensitive environments or for vulnerable populations.
3. **Comparison with Existing Technologies:** The paper lacks a thorough comparison with existing eye-gesture control technologies. While the accuracy of the proposed system is commendable, readers would benefit from understanding how this system stacks up against other similar solutions in terms of performance, usability, and practicality. A comparative analysis would provide valuable context for evaluating the system's novelty and effectiveness.
4. **Face Landmark Detection Method:** The method employed for face landmark detection is not original to the authors and relies on existing algorithms. If the authors choose to apply established methods, it would be beneficial to include more detailed design elements and interface considerations specific to this application. For further insights and ideas, the authors may refer to the following works:
  - Chen, H, et.al., 2023 (Ref 1): Chen, H., Zendehdel, N., Leu, M.C. and Yin, Z., 2023. Real-time human-computer interaction using eye gazes. *Manufacturing Letters*, *35*, pp.883-894.
  - Chen, H, et.al., 2022 (Ref 2): Chen, H., Zendehdel, N., Leu, M.C. and Yin, Z., 2022. Multi-Modal Fine-Grained Activity Recognition and Prediction in Assembly. *Research Square Platform LLC*.
  - Chen H, et.al., 2024 (Ref 3): Chen, H., Zendehdel, N., Leu, M.C. and Yin, Z., 2024. Fine-grained activity classification in assembly based on multi-visual modalities. *Journal of Intelligent Manufacturing*, *35*(5), pp.2215-2233.

**Minor Points:**

1. **Dataset Diversity:** The paper provides limited information on the diversity of the dataset used for testing the system. Given the importance of generalizability in AI systems, it is essential to ensure that the dataset is representative of various demographics and usage conditions. Please Include more details on the dataset's composition and how it impacts the system's performance across different scenarios.

**Strengths:**

- The system's high accuracy rate of over 99.63% is a significant achievement, highlighting its potential for practical applications.
- The use of accessible and widely recognized libraries such as OpenCV, mediapipe, and pyautogui is a commendable choice, making the technology easier to replicate and adapt by others.
- The potential applications of this technology, particularly in aiding individuals with physical impairments and in critical fields like military and rescue operations, are well-justified and promising.

**Overall Evaluation:**

 The study design is generally appropriate, and the work is technically sound. However, the paper could benefit from a more in-depth discussion of long-term usability, privacy concerns, and a comparison with existing technologies. Sufficient details of the methods are provided to allow replication, though additional information on dataset diversity would be advantageous. The conclusions are well-supported by the results, but the inclusion of the aforementioned points would significantly strengthen the paper.

Is the work clearly and accurately presented and does it cite the current literature?

Yes

If applicable, is the statistical analysis and its interpretation appropriate?

Partly

Are all the source data underlying the results available to ensure full reproducibility?

Partly

Is the study design appropriate and is the work technically sound?

Partly

Are the conclusions drawn adequately supported by the results?

Partly

Are sufficient details of methods and analysis provided to allow replication by others?

Partly

Reviewer Expertise:

Computer Vision, Artificial Intelligence, AI, Machine Learning, Deep Learning, Human-Computer Interaction (HCI), Eye Gaze Estimation, Gesture Recognition

I confirm that I have read this submission and believe that I have an appropriate level of expertise to confirm that it is of an acceptable scientific standard, however I have significant reservations, as outlined above.

## References

[1] : Real-time human-computer interaction using eye gazes. *Manufacturing Letters* .2023;35: 10.1016/j.mfglet.2023.07.024 883-894 10.1016/j.mfglet.2023.07.024

[2] : Real-Time Multi-Modal Human–Robot Collaboration Using Gestures and Speech. *Journal of Manufacturing Science and Engineering* .2022;144(10) : 10.1115/1.4054297 10.1115/1.4054297

[3] : Fine-grained activity classification in assembly based on multi-visual modalities. *Journal of Intelligent Manufacturing* .2024;35(5) : 10.1007/s10845-023-02152-x 2215-2233 10.1007/s10845-023-02152-x

# Response

**Published:** 2024-10-20

**Authors:** Nachaat Mohamed

**Affiliations:** HLS, Rabdan Academy, Abu Dhabi, Abu Dhabi, United Arab Emirates

**Thanks a lot Dr. Haodong Chen**

 We will address this concern by discussing the potential for eye strain and the need for breaks during prolonged use. We will also suggest future enhancements that could mitigate these issues.

 We will address privacy concerns by explaining how data is anonymized and secured, in compliance with relevant data protection regulations.

 We will add a comparative analysis of existing eye-gesture control technologies, highlighting the differences in accuracy, ease of implementation, and other performance metrics.

 We will acknowledge the use of established face landmark detection algorithms and explain the specific adaptations made for our system to improve performance.

 We will elaborate on the dataset used, specifying participant details, diversity, and dataset composition to ensure the robustness of the algorithm is clear.

# Response

**Published:** 2024-12-04

**Authors:** Nachaat Mohamed

**Affiliations:** HLS, Rabdan Academy, Abu Dhabi, Abu Dhabi, United Arab Emirates

Thank you for this valuable observation. We have added a discussion on the long-term usability and potential comfort issues associated with prolonged use of the eye-gesture system. Specifically, we addressed concerns about eye strain and proposed potential mitigation strategies, such as incorporating adaptive interface designs and periodic usage breaks. These additions can be found in the revised Discussion section.

 We appreciate this important suggestion. To address this, we have included a detailed discussion on privacy and data protection measures in the Discussion section. The revised text highlights our strict data anonymization protocols, encryption methods, and adherence to international privacy standards, such as GDPR. These measures ensure that user privacy is safeguarded during data collection and processing.

 Thank you for pointing this out. We have expanded the Related Work section to include a comparison with existing eye-gesture control technologies. The updated section highlights the advantages of our system, including its higher accuracy (99.63%) compared to reported ranges (95%–99%), and its reliance on accessible tools like OpenCV and PyAutoGUI, which eliminate the need for specialized hardware. This comparison emphasizes the practical and cost-effective nature of our approach.

 We acknowledge this comment and have clarified in the Methodology section how our system builds upon existing face landmark detection algorithms (e.g., MediaPipe) by incorporating customized calibration techniques. These adaptations optimize the system’s performance for real-time applications and diverse user conditions, addressing specific challenges such as varying facial structures and dynamic lighting environments. The revisions outline the unique contributions of these enhancements.

 Thank you for highlighting this. We have revised the Methodology section to provide detailed information about the dataset. It comprises 20,000 gesture instances collected from 100 volunteers representing diverse demographics, including variations in age, gender, and ethnicity. The diversity of the dataset was intentionally designed to ensure the system's generalizability and robustness in real-world applications.

# Reviewer report: Reviewer response for version 1

**DOI:** 10.5256/f1000research.158833.r285546

**Published:** 2024-07-31

**Authors:** Zakariyya Abdullahi Bature

**Affiliations:** 1 King Mongkut's University of Technology Thonburi, Bangkok, Thailand

This paper demonstrated AI-based eye-gesture recognition for computer system control. The author tries to capture the raw eye movement and translates it into human action using an AI-based algorithm. As it stands, I have a few concerns/questions to recommend this paper for indexed.

 1. In the abstract and the method, this work described the tools used but partially described the algorithms, and how they solve the problems compared to the previous works. Moreover, it does not accurately describe the exact problems of the existing works. How the proposed method captures and converts the eye movement into actions. 

 2. The details of the dataset are poorly explained. Even though the author has provided the link to the dataset, but is poorly explained and labeled. Details such as the number of participants in the dataset contained, number of the gestures, repetitions, and frame numbers are needed to assess the robustness of the algorithms.

 3. Details of the presentation of the results are needed in terms of the number of gestures. Please use some of the visualizing tools such as the confusion matrix, bar chart, etc. to visualize the recognition performance of your proposed method on each eye gesture.

 4. It is very difficult to conclude the robustness of your method from the results of a single dataset. Please, use publicly available datasets to validate your proposed method and compare the results of the datasets with state-of-the-art methods.

 5. Please state the drawback of your proposed method in the conclusion.

Is the work clearly and accurately presented and does it cite the current literature?

Partly

If applicable, is the statistical analysis and its interpretation appropriate?

Partly

Are all the source data underlying the results available to ensure full reproducibility?

Partly

Is the study design appropriate and is the work technically sound?

Yes

Are the conclusions drawn adequately supported by the results?

Partly

Are sufficient details of methods and analysis provided to allow replication by others?

Partly

Reviewer Expertise:

Machine Learning with interests in Computer Vision, Digital Image Processing, Feature Selection Methods, Optimization with their application to Eye Tracking, Human Gesture Recognition, Signal Processing, Biometrics Identification and related topics

I confirm that I have read this submission and believe that I have an appropriate level of expertise to confirm that it is of an acceptable scientific standard, however I have significant reservations, as outlined above.

# Response

**Published:** 2024-10-20

**Authors:** Nachaat Mohamed

**Affiliations:** HLS, Rabdan Academy, Abu Dhabi, Abu Dhabi, United Arab Emirates

**Thanks a lot Dr. Zakariyya Abdullahi**

 We will enhance the explanation of the algorithm, comparing it with prior works and highlighting how our system addresses the gaps. This will clearly detail how the eye gestures are translated into commands, emphasizing the novelty of our approach.

 We will elaborate on the dataset used, specifying participant details, diversity, and dataset composition to ensure the robustness of the algorithm is clear.

 We will include visual Confusion Matrix at the end of the results section **,** to better illustrate the system's performance and its effectiveness across various gestures.

 We will clarify that the dataset used in this study was collected from 100 volunteers, ensuring a diverse representation in terms of age, gender, and ethnicity. This dataset, comprising 20,000 gesture instances, was instrumental in achieving a high accuracy rate of 99.63%. However, we acknowledge the importance of further validating the system using publicly available datasets to enhance generalizability. We are currently exploring the integration of external datasets, such as those from Dryad, to perform comparative analysis and further assess the robustness of our model in different scenarios.

 We will explicitly state the potential limitations of the method, including the challenges of using the system in low-light conditions and potential issues with glasses or contact lenses.

# Response

**Published:** 2024-12-04

**Authors:** Nachaat Mohamed

**Affiliations:** HLS, Rabdan Academy, Abu Dhabi, Abu Dhabi, United Arab Emirates

Thank you for highlighting this point. We have revised the manuscript to provide a detailed description of the algorithm. The updated text includes an explanation of how the system uses machine learning models integrated with OpenCV and PyAutoGUI to capture eye movements and translate them into actionable commands. Additionally, we have added a comparison with prior works, emphasizing the improvements in speed, accuracy, and generalizability. These changes can be found in the revised **Methodology** and **Related Work** sections.

 We appreciate this observation and have updated the manuscript to include comprehensive details about the dataset. Specifically, the dataset comprises 20,000 gesture instances collected from 100 volunteers, each representing a diverse range of demographics. Each participant performed 10 distinct gestures, repeated 20 times. These details, along with the methodology for dataset collection and preprocessing, are now clearly outlined in the **Methodology** section.

 Thank you for this suggestion. We have incorporated visual representations, including a confusion matrix and bar charts, to present the system's performance across the 10 distinct gestures. These additions are now included in the **Results** section, providing a clearer understanding of classification accuracy, potential misclassifications, and overall performance metrics.

 We acknowledge the importance of validating the system with publicly available datasets. While the primary dataset used in this study was collected from 100 volunteers to ensure diversity and robustness, we are actively exploring external datasets, such as those from Dryad, for further validation and comparison with other state-of-the-art methods. This effort will be part of our future work and has been discussed in the revised **Results** and **Conclusion** sections.

 Thank you for this recommendation. We have updated the **Conclusion** section to explicitly discuss the system's limitations. These include minor accuracy reductions in scenarios involving reflective glasses (98.9%) and the need for further validation with publicly available datasets to enhance generalizability. We believe this addition provides a more balanced and complete assessment of the system.
